# Harnessing genetic resistance to rusts in wheat and integrated rust management methods to develop more durable resistant cultivars

**DOI:** 10.3389/fpls.2022.951095

**Published:** 2022-10-14

**Authors:** Johannes Mapuranga, Na Zhang, Lirong Zhang, Wenze Liu, Jiaying Chang, Wenxiang Yang

**Affiliations:** College of Plant Protection, Technological Innovation Center for Biological Control of Plant Diseases and Insect Pests of Hebei Province, Hebei Agricultural University, Baoding, China

**Keywords:** wheat, stem rust, stripe rust, leaf rust, wheat rust management, genetics and resistance

## Abstract

Wheat is one of the most important staple foods on earth. Leaf rust, stem rust and stripe rust, caused by *Puccini triticina*, *Puccinia* f. sp. *graminis* and *Puccinia* f. sp. *striiformis*, respectively, continue to threaten wheat production worldwide. Utilization of resistant cultivars is the most effective and chemical-free strategy to control rust diseases. Convectional and molecular biology techniques identified more than 200 resistance genes and their associated markers from common wheat and wheat wild relatives, which can be used by breeders in resistance breeding programmes. However, there is continuous emergence of new races of rust pathogens with novel degrees of virulence, thus rendering wheat resistance genes ineffective. An integration of genomic selection, genome editing, molecular breeding and marker-assisted selection, and phenotypic evaluations is required in developing high quality wheat varieties with resistance to multiple pathogens. Although host genotype resistance and application of fungicides are the most generally utilized approaches for controlling wheat rusts, effective agronomic methods are required to reduce disease management costs and increase wheat production sustainability. This review gives a critical overview of the current knowledge of rust resistance, particularly race-specific and non-race specific resistance, the role of pathogenesis-related proteins, non-coding RNAs, and transcription factors in rust resistance, and the molecular basis of interactions between wheat and rust pathogens. It will also discuss the new advances on how integrated rust management methods can assist in developing more durable resistant cultivars in these pathosystems.

## 1 Introduction

Wheat is a major food crop, with a total area of more than 219 million hectares and an annual production of more than 760 million tonnes (Faostat, 2020). It provides around 20% of the world human population’s daily caloric needs (Faostat, 2016). Every year, fungi and insects devastate worldwide wheat yield to the tune of 21.5% (Savary et al., 2019). Biotrophic pathogenic fungi cause rust diseases, which are among the most economically important diseases affecting wheat production. Stem rust (black rust), leaf rust (brown rust), and stripe rust (yellow rust), all caused by the rust pathogens *Puccinia graminis* f. sp. *tritici* (*Pgt*), *Puccinia triticina* (*Pt*), and *Puccinia striiformis* f. sp. *tritici* (*Pst)*, respectively, continue to endanger worldwide wheat production on a year-round basis (McIntosh et al., 1995; Dean et al., 2012; Hafeez et al., 2021; Mapuranga et al., 2022a). Stem rust caused by *Pgt* is often regarded as one of the most destructive wheat rust disease because if not controlled, it can wipe all the crops within a short space of time (Singh et al., 2011). A stripe rust infection may develop at any moment throughout the plant’s life cycle, from the one-leaf stage until the time of maturity, as long as the plants are still growing. Over 60 nations have reported cases of wheat stripe rust, which may be found on every continent except Antarctica (Savary et al., 2019). Recently, catastrophic stripe rust outbreaks in key wheat-producing nations resulted in large yield losses. *Pt* primarily infects wheat leaves at various developmental stages as well as leaf sheath and glumes (Figlan et al., 2020). *Pt* significantly impedes the production of wheat, causing high yield losses (Samborski, 1985). Leaf rust occurs in many temperate wheat-producing areas due to its adaptation to a wide range of environments, causing yield losses of up to 70%. (Marasas et al., 2003; Huerta-Espino et al., 2006; Aktar-Uz-Zaman et al., 2017). Resistance to leaf rust, stripe rust, and stem rust is conferred by a diverse set of genes designated *Lr, Yr*, and *Sr*, respectively (Zhang et al., 2020b).

Wheat genome sequences from diploid, tetraploid, and even hexaploid wheats are being sequenced and annotated frequently and rapidly making it possible to discover and characterize new resistance genes that can be used by wheat breeders to improve wheat resistance to multiple pathogens (Appels et al., 2018, Ramírez-González et al., 2018; Bailey-Serres et al., 2019; Gerten et al., 2020). Disease resistance in natural plant-pathogen interactions can be divided broadly into resistance that is expressed against all isolates of a pathogen (non-race specific resistance) and resistance that is expressed only against specific pathogen phenotypes (race specific resistance). Race-specific resistance confers mostly ample resistance to some pathogens and not others, and it is conferred by single resistance (*R*) (major effect) genes and is comparatively inherited. Non-race specific resistance confers partial resistance; is independent of specific avirulence (*Avr*) genes and also allows infection but lowers pathogen proliferation. The two different types of resistance can be conferred by the same genes, in which a host gene may confer hypersensitive resistance to some isolates and a rate-reducing resistance to others (Maurya et al., 2021).

More than 100, 80 and 66 resistance genes have been found for wheat leaf rust, stripe rust, and stem rust, respectively, with the bulk of them having been already mapped on wheat chromosomes using DNA markers (McIntosh et al., 2017; Hafeez et al., 2021; Kumar et al., 2022; Yu et al., 2022). Flor proposed a gene-to-gene relationship for each resistance gene using the *Linum-Melampsora* host-pathogen approach (Flor, 1955). According to this hypothesis, the function of each resistance gene is dependent on a corresponding pathogen avirulence gene. Therefore, in the absence of this avirulence gene, the resistance gene may not confer resistance (Dodds et al., 2006). More than 30 wheat resistance genes have been cloned in order to better understand the nature of their gene products, which has improved our knowledge of the molecular mechanism of resistance to leaf rust, stem rust, and stripe rust. More than half of the *R* genes in plants, including the leaf rust, stem rust, and stripe rust genes in wheat, are members of the nucleotide-binding domain and leucine-rich repeat (NLR) family. Other cloned *R* genes encode different protein families including ATP-binding cassette (ABC) and steroidogenic acute regulatory protein-related lipid transfer (START), etc. The never-ending quest for higher yields while simultaneously improving quality does not come without its difficulties. Increased pathogen emergence has resulted from the loss of wheat genetic variety due to the pursuit of elite, high-performing cultivars. As a result of this drop in genetic diversity, diseases now endanger the global wheat supply (Figueroa et al., 2018). We believe that future genomics research and application will aid in the breeding of wheat varieties with more permanent resistance than what is now available, and that we may finally win the never-ending arms race between host and pathogen.

However, despite great progress in the management of wheat diseases due to technological and scientific innovation, plant diseases continue to pose major threats to global wheat production (Jeger et al., 2021; Prasad et al., 2021). Pathogens and respective diseases can be directly or indirectly affected by climate change (Juroszek et al., 2020). Temperature, relative humidity, rainfall, photoperiod, wind direction and speed, date of sowing, and maturity of crops, all influence the growth, multiplication, pathogenesis, dissemination, and survival of plant pathogens (Prasad et al., 2021). Relative humidity, ambient temperature, and precipitation have the greatest influence on the outcome of a particular host-pathogen interaction, pathogens dissemination, and survival (Prank et al., 2019). Therefore, predicting the likely consequences of climate change on the host, pathogen, their interaction, population dynamics, agro-ecosystem community structure, and micro-evolutionary developments is required before considering the effects of changing climate on particular crop disease (Prasad et al., 2021). This review gives a succinct and up-to-date overview of rust resistance, particularly race-specific and non-race specific resistance, the role of pathogenesis-related proteins, non-coding RNAs, and transcription factors in conferring rust resistance. It will also discuss the molecular basis of interactions between wheat and rust pathogens and how integrated rust management approaches can aid in developing more durable resistance cultivars.

## 2 Pathogen perception

It is common for rust to get access to the plant core by directly penetrating the leaf or stem surfaces and entering the plant *via* leaf stomata. During the course of an infection, plants are equipped with the ability to detect the presence of pathogens at many levels (Jones and Dangl, 2006), and as a consequence, the host defense system is activated. Pattern-recognition receptors (PRRs) on the cell membranate detect pathogen-associated molecular patterns (PAMPs) and trigger PAMP-triggered immunity (PTI). Establishing a dynamic parasitic relationship between the biotrophic fungi and the host is the foundation for the development of the pathogen in the host plant. In order to infect the host plant successfully, rust pathogens suppress PTI components by secreting virulence factors called effectors into the host cells through the haustoria and hyphae resulting in effector-triggered susceptibility (ETS) (Martel et al., 2021; Mapuranga et al., 2022a; Mapuranga et al., 2022b). The plants in response developed a second layer of innate immunity known as effector-triggered immunity (ETI), in which the plant resistance proteins recognize corresponding avirulence factors and set off a powerful defensive response. As originally proposed by Jones and Dangl, the PTI-ETS-ETI cycle continues and is portrayed as a zig-zag model (Jones and Dangl, 2006).

During ETI, the host immune system is activated by NLRs’ detection of effector molecules secreted by the pathogen. This detection can occur either directly or indirectly, with the NLR (also known as the guard) recognizing the effector-mediated alteration of a host pathogenicity target or a decoy of this target (also known as the guardee) (van der Hoorn and Kamoun, 2008; Dodds and Rathjen, 2010). In NLR proteins, the C-terminal leucine-rich repeats provide the guarding function, while the N-terminal nucleotide-binding and coiled-coil or TIR domains confer the signaling capacity. The guard model unraveled a useful framework for understanding the molecular mechanisms and evolution of plant resistance genes. All three interactors; guard, guardee and effector are subject to diversifying selection, but for the guardee, this can be constrained by the requirement to maintain cellular function. The decoy pathogenicity target may be integrated into the NLR itself in certain circumstances. Sensor NLRs frequently collaborates with a second helper NLR, which initiates downstream signaling upon sensor NLR activation (Cesari et al., 2014; Kourelis and van der Hoorn, 2018). The wheat *Lr10* locus contains two NLR-encoding genes that are necessary for resistance (Loutre et al., 2009). Race-specific leaf rust disease resistance gene *Lr14a* encodes a membrane-localized protein containing twelve ankyrin (ANK) repeats with structural resemblances to Ca^2+^-permeable non-selective cation channels. The ANK domain could be a direct target of pathogen effectors and *Lr14a* indirectly recognizes the AvrLR14A (Kolodziej et al., 2021). The wheat stripe rust resistance gene *YrU1* encodes a coiled-coil nucleotide binding site leucine-rich repeat (CC-NBS-LRR) protein with N-terminal ankyrin-repeat and C-terminal WRKY domains immune receptors (Wang et al., 2020b). The ANK domain of YrU1 is derived from ANK-transmembrane proteins and possibly serves as a decoy for pathogen effectors (Wang et al., 2020b). Self-association of the N-terminal of NLR proteins of *Sr33*, *Sr50* plays a crucial role in triggering downstream immune signals (Cesari et al., 2016). *Sr33* and *Sr35* can induce an effector-independent cell death response *in planta* (Cesari et al., 2016), and the nominal defense signaling component is the N-terminal CC domain and the dimerization of this domain is required for signaling. *Sr62* is also a pathogenicity target guarded by an NLR (Yu et al., 2022).

## 3 Genetics of wheat resistance to rusts

Many attempts have been made to extract rust-resistant genes in cereal plants and know how to effectively deploy them for long-term disease management. Genetic resistance may be effective and chemical-free. Many efforts are being devoted at extracting rust-resistant genes in wheat and knowing how to best deploy them for long-term resistance since genetic resistance can provide effective and chemical-free disease control (Ellis et al., 2014). Furthermore, closely related nonhost species are increasingly being used to uncover new sources of resistance (Kawashima et al., 2016). However, although host genotype resistance and fungicide application are the most generally utilized approaches for controlling wheat rusts, effective agronomic approaches are required to reduce disease management costs and increase wheat production sustainability. A greater knowledge of the spatial and temporal heterogeneity in the structure of wheat rust growth must aid in more effective and long-term disease control (Naseri and Sharifi, 2019). For example, an integration of earlier disease onset, later planting and maturation, lower cultivar resistance, warmer winter, and even colder and wetter days throughout the autumn-winter-spring period exacerbated leaf and stripe rust outbreaks in wheat crops harvests (Naseri and Marefat, 2019; Naseri and Sasani, 2020). These substantial linkages also highlighted the potential of increasing resistance levels in wheat cultivars by selecting the correct planting date to limit disease development under optimal climatic conditions. However, there is limited knowledge about a combined interaction of climate (relative humidity and temperature), disease (onset and severity), genotype (maturity and resistance), and planting date to predict intensity of wheat leaf rust (Naseri and Sasani, 2020).

Plant genetic resistance to diseases caused by biotrophic fungi is essential for breeding crops worldwide because it provides innovative strategies for disease control (Jiquel et al., 2021). There are two types of genetic resistance to rust infection in wheat namely, race-specific resistance and non-race specific resistance. Over 200 resistance genes to fungal rusts have been genetically identified with the majority conferring race-specific resistance (McIntosh et al., 1995; McIntosh et al., 2013; McIntosh et al., 2017). So far in wheat, more than 100, 80 and 66 genes for resistance against leaf rust, stripe rust, and stem rust respectively, have already been identified and designated globally, and are distributed on all the 21 wheat chromosomes (McIntosh et al., 2017; Hafeez et al., 2021; Jan et al., 2021; Kumar et al., 2022). More than 100 leaf rust resistance genes (~50% derived from wild progenitor and non-progenitor species) have been identified, and only eleven of these have been cloned so far, including *Lr1* (Cloutier et al., 2007), *Lr9* (Wang et al., 2022), *Lr10* (Feuillet et al., 2003), *Lr13* (Hewitt et al., 2021; Yan et al., 2021), *Lr14a* (Kolodziej et al., 2021), *Lr21* (Huang et al., 2003), *Lr22a* (Thind et al., 2017), *Lr34/Yr18/Sr57* (Krattinger et al., 2009), *Lr42* (Lin et al., 2022), *Lr58* (Wang et al., 2022), and *Lr67/Yr46/Sr55* (Moore et al., 2015) (**Table 1**). Only ten of the stripe rust resistance genes including *Yr5/YrSp* (Marchal et al., 2018)*, Yr7* (Marchal et al., 2018)*, Yr10* (Liu et al., 2014), *Yr15* (Klymiuk et al., 2018)*, Yr27* (Athiyannan et al., 2022)*, Yr36* (Fu et al., 2009; Gou et al., 2015), *Yr18* (Krattinger et al., 2009)*, YrU1* (Wang et al., 2020b), *Yr46* (Moore et al., 2015) and *YrAS2388R* (Zhang et al., 2019) have been cloned so far (**Table 1**). Till date, 66 distinct stem rust resistance genes on 59 loci have been designated in wheat, and just over half of them are from bread wheat, while the remainder were introgressed into wheat from wild and related spp. It was recently reported that, eight *Sr* genes are from domesticated wheat *Triticum* spp., eleven are from *Aegilops* spp., four are from *Secale cereale*, four are from wheat grass *Thinopyrum* spp., and one is from the grass *Dasypyrum villosum* (Hafeez et al., 2021; Gaurav et al., 2022; Yu et al., 2022). 15 of the 66 designated *Sr* genes have been cloned so far, including *Sr13* (Zhang et al., 2017b; Gaurav et al., 2022), *Sr21* (Chen et al., 2018), *Sr22* (Steuernagel et al., 2016), *Sr26* (Zhang et al., 2021a), *Sr33* (Periyannan et al., 2013), *Sr35* (Saintenac et al., 2013), *Sr45* (Steuernagel et al., 2016), *Sr46* (Arora et al., 2019), *Sr50* (Mago et al., 2015), *Sr55*/*Lr67* (Moore et al., 2015), *Sr57*/*Lr34*  (Krattinger et al., 2009), *SrTA1662* (Arora et al., 2019), *Sr60* (Chen et al., 2020), *Sr61* (Zhang et al., 2021a) and *Sr62* (Yu et al., 2022). The continuous emergence of new races of rust pathogens complicates the maintenance of effective sources of genetic resistance in the field and emphasizes the difficulties inherent in controlling these diseases purely *via* genetic resistance (Ellis et al., 2014).

**Table 1:** A summary of cloned *Lr, Sr*, and *Yr* genes for leaf rust, stem rust, and stripe rust resistance, respectively.

Gene cloned	Chromosome position	*R*-gene product	*R*-gene class	Cloning Technique	References
*Lr1*	5DL	A coiled coil (CC), nucleotide binding site (NBS), leucine rich-repeat (LRR) protein of 1344 amino acid residues	All-stage resistance	Map-based cloning	(Cloutier et al., 2007)
*Lr9*	6BL	An unusual tandem kinase fusion protein of 1167 amino acid residues with an N-terminal tandem kinase domain followed by a von Willebrand factor A (vWA) domain and a Vwaint domain in the C-terminal.	All-stage resistance	MutIsoSeq	(Wang et al., 2022)
*Lr10*	1AS	A 919 amino acid residues CC-NBS-LRR protein with an N-terminal domain	All-stage resistance	Map-based cloning	(Feuillet et al., 2003)
*Lr13/Ne2*	2BS	A CC-NBS-LRR protein of 1073 amino acid residues	High temperature adult plant resistance. Exhibits pleiotropic effects on hybrid necrosis	MutRenSeq	(Hewitt et al., 2021; Yan et al., 2021)
*Lr14a*	7BL	A protein of 779 amino acids with an N-terminal domain containing 12 ANK repeats followed by six predicted transmembrane helices	All-stage resistance	MutChromSeq	(Kolodziej et al., 2021)
*Lr21*	1DL	A 1080 amino acid residues protein having a conserved NBS domain with 13 LRRs and distinct 151-amino acid sequence	All-stage resistance	Map-based cloning	(Huang et al., 2003)
*Lr22a*	2DS	A 912 amino-acid immune receptor protein similar to *Arabidopsis* RPM1	Adult -stage resistance N-terminal amino acids of RPM1 interact with the RPM1-interacting protein 4, resulting in a HR	Map-based cloning and TACCA	(Thind et al., 2017)
*Lr34/Yr18/Sr57*	7DS	A 1401 amino acid ABC transporter with similarity to pleiotropic drug resistance subgroup of ABC transporters	Adult plant resistance Possible toxin transporter. *Lr34res* increases anti-fungal phenylpropanoid diglyceride	Map-based cloning	(Krattinger et al., 2009)
*Lr42*	IDS	A typical CC-NBS-LRR protein of 920 amino acid residues and is likely to be a singleton or helper NLR not sensor NLR	All-stage resistance	BSR-Seq mapping	(Lin et al., 2022)
*Lr58*	2BL	An unusual tandem kinase fusion protein of 1167 amino acid residues with an N-terminal tandem kinase domain followed by a von Willebrand factor A (vWA) domain and a Vwaint domain in the C-terminal.	All-stage resistance	MutIsoSeq	(Wang et al., 2022)
*Lr67/Yr46/Sr55*	4DL	A 514-amino acid hexose transporter with 12 trans-membrane helices	Adult plant resistance or HTAP restistance. Negatively regulates hexose transporter	Map-based cloning	(Moore et al., 2015)
*Sr13*	6AL	A CC-NBS-LRR protein of 948 amino acid residues	All-stage resistance	Map-based cloning	(Zhang et al., 2019)
*Sr21*	1DS	A CC-NBS-LRR protein of 1624 amino acid residues	All-stage resistance	Map-based cloning	(Chen et al., 2018)
*Sr22*	7AL	A 941 amino acid residues CC-NBS-LRR protein	All-stage resistance	MutRenSeq	(Steuernagel et al., 2016)
*Sr26*	6A	A CC-NBS-LRR protein of 880 amino acid residues	All-stage resistance	MutRenSeq	(Zhang et al., 2021a)
*Sr33*	1DS	A CC-NBS-LRR protein of 961 amino acid residues	All-stage resistance	Map-based cloning	(Periyannan et al., 2013)
*Sr35*	3AL	A CC-NBS-LRR protein of 919 amino acid residues	All-stage resistance	Map-based cloning	(Saintenac et al., 2013)
*Sr45*	1DS	A CC-NBS-LRR protein of 1230 amino acid residues	All-stage resistance	MutRenSeq	(Steuernagel et al., 2016)
*Sr46*	2DS	A CC-NBS-LRR protein of 924 amino acid residues	All-stage resistance	AgRenSeq	(Arora et al., 2019)
Sr50*	1D	A CC-NBS-LRR protein of 956 amino acid residues	All-stage resistance	Map-based cloning	(Mago et al., 2015; Chen et al., 2017b)
*Sr60* (*WKS2*)	5AS	A tandem protein kinase of 724 amino acid residues	All-stage resistance	Map-based cloning	(Chen et al., 2020)
*Sr61*	6E	A protein of 880 amino acid residues with CC, NB-ARC domains and LRR motifs	All-stage resistance	MutRenSeq	(Zhang et al., 2021a)
*Sr62*	1S^sh^	A tandem protein kinase of 740 amino acid residues	All-stage resistance	AgRenSeq	(Yu et al., 2022)
*SrTA1662*	1DS	A CC-NBS-LRR protein	All-stage resistance	AgRenSeq	(Arora et al., 2019)
*YrAS2388R*	4DS	A CC-NBS-LRR protein of 1068 amino acid residues	All-stage resistance	Map-based cloning	(Zhang et al., 2019)
Yr5a(Yr5), Yr5b (YrSP)**	2BL	NBS-LRR proteins of 1522 and 876 amino acid residues, respectively, with a distinct N-terminal zinc-finger BED domain	All-stage resistance	MutRenSeq	(Marchal et al., 2018)
*Yr7*	2BL	A NBS-LRR protein of 1586 amino acid residues with a distinct N-terminal zinc-finger BED domain	All-stage resistance	MutRenSeq	(Marchal et al., 2018)
*Yr10* (*Yr10cg*) ***	1BL	A CC-NBS-LRR protein of 824 amino acid residues, possessing 11 imperfect LRR units, P-loop, kinase-2a and kinase-3a conserved domains,	All-stage resistance	Map-based cloning	(Liu et al., 2014)
*Yr15/YrG303/YrH52*	1BS	Tandem kinase-pseudokinase (TKP) of 665 amino acid residues.	All-stage resistance	Map-based cloning	(Klymiuk et al., 2018)
*Yr27*	2BS	A highly variable NLR of 1072 amino acid residues with a variable stretch withing its LRR domain that might serve for defining recognition specificity. Allelic to leaf rust resistance gene *Lr13*	All-stage resistance	Long-read genome sequencing	(Athiyannan et al., 2022)
*Yr36*	6BS	A protein of 645 amino acid residues with a combination of serine/threonine kinase START domains	Adult plant resistance	Map-based cloning	(Fu et al., 2009; Gou et al., 2015)
*YrU1*	5AL	An ANK-NLR-WRK protein of 1576 amino acid residues with N-terminal ANK repeat and C-terminal WRKY domains	All-stage resistance Homodimerization of the CC and ANK domains resulting in transduction of immune signals	Map-based cloning	(Wang et al., 2020b)

Lr leaf rust, Sr stem rust, Yr stripe rust.

*Sr50 is located on 1D in wheat but in rye it is on 1RS.

**Yr5a and Yr5b are alleles.

***The cloned gene originally proposed to be Yr10, has been found to encode a different resistance gene, Yr10cg (Yuan et al., 2018).

### 3.1 Race-specific resistance

Race-specific resistance is also known as qualitative resistance, vertical resistance, specific resistance, major gene resistance, monogenic resistance, actual resistance, all-stage resistance or whole resistance. Since it is unique to particular pathogen races and is susceptible to other pathogen races, this kind of resistance is vertical resistance. It is often achieved by the improvement of a hypersensitive pinpoint fleck reaction. It is referred to as major gene resistance because it is governed by a significant gene (Maurya et al., 2021). It is common to discover race-specific resistance during the seedling or adult development stages when various resistance responses to infection are seen. It is often inherited qualitatively, has a limited life span, and is readily defeated by more aggressive races of rust fungus that emerge (Maurya et al., 2021). It is common to discover race-specific resistance during the seedling or adult development stages when various resistance responses to infection are seen. It is often inherited qualitatively, has a limited life span of 3 – 5 years, and is readily defeated by more aggressive emerging races of rust pathogens (Maurya et al., 2021). It is referred to as whole/actual resistance because the resistance manifests itself in the form of complete suppression of disease symptoms improvement. Race-specific resistance genes typically follow the conventional gene-for-gene paradigm, in which resistance is determined by a particular genetic interaction between a host resistance gene and its corresponding avirulence gene (Periyannan et al., 2017). In most cases, race-specific resistance manifests itself as hypersensitive response, which is characterized by rapid cell death that occurs at the interface between fungal haustoria and host cells in the epidermal and mesophyll layers. A total of 66 genes/alleles at 59 loci confer resistance to *Pgt* in wheat, and of the 59 cataloged loci, 53 are expressed at all growth stages, and six confer adult plant resistance.

Different *R* genes influence the development of distinct resistance phenotypes or infection types in different cultivars. Examples of such responses include wheat lines with *Lr3* showing well marked hypersensitive flecks, while lines with *Lr2a* showed only very faint flecks that were difficult to distinguish from the background grain coloration (Bolton et al., 2008). In addition to these race-specific resistance responses, those conditioned by wheat lines with *Lr3ka, Lr3bg*, and *Lr11* showed tiny uredinia surrounded by chlorosis, while those conditioned by wheat lines with *Lr16* showed trivial uredinia bounded by necrosis. *Lr14a*-containing lines displayed a wide range of mesothetic resistance responses with different portions of hypersensitive flecks and leaf rust uredia. Moreover, the *Lr14a* gene activity has been classified as temperature sensitive (Kolodziej et al., 2021). *Lr* genes that are unique to a certain race are effective in seedling plants and continue to be functional in mature plants. Although certain genes, such as *Lr12* and *Lr13*, and *Lr22a* are responsible for conditioning resistance in young plants, the resistance conditioned by these genes is best manifested in mature plants. In wheat lines that have combinations of resistance genes, the gene that confers the most resistance to the most severe infection type is epistatic to the gene that confers the least resistance to the least severe infection type (Bolton et al., 2008).

Many studies have been conducted to characterize temperature-responsive resistance genes using various pre and post inoculation conditions, but the influence of temperature change has seldom been examined. However, temperatures change regularly in nature, and the influence of this on resistance requires additional exploration. Understanding how temperature variations impact resistance might lead to the breeding of more stable pathogen resistance in wheat (Bryant et al., 2014). Several studies predicted the severity of wheat leaf rust epidemics using a number climate, crop and disease variables and different disease management descriptors such as disease-cycle, host-growth stages, planting date and cultivar resistance (Rao et al., 1990; Rossi et al., 1997; Moschini and Pérez, 1999; Räder et al., 2007; Naseri and Mousavi, 2013; Naseri, 2014a; Naseri, 2014b; Savary et al., 2015; Naseri and Marefat, 2019; Naseri and Sharifi, 2019; Naseri and Sasani, 2020; Naseri and Sabeti, 2021). Recently, two studies established interrelationships among disease onset date, leaf rust severity area under disease progressive curve, planting time, maturity time, relative humidity of greater than 60% mean six-monthly and temperature range of 5-25°C (Naseri and Sasani, 2020; Naseri and Sabeti, 2021). Such a close relationship between wheat maturation and planting date as wheat leaf rust predictors appears significant, because planting date has also been considered a primary disease management operation in a wide range of agricultural crops (Moschini and Pérez, 1999; Naseri, 2013; Naseri, 2014a; Naseri, 2014b; Naseri and Sharifi, 2019). Furthermore, these findings may enhance breeding for more resistant genotypes given that a combination of climate, maturity and planting date would be studied for screening wheat varieties (Naseri and Sasani, 2020). Further research is required to explore these links in diverse geographical locations with varying host and pathogen genotypes, and climatic conditions environmental circumstances. These new findings suggested that current predicting models should be improved by including dates of maturation and sowing as well as wheat resistance in addition to rainfall-temperature-wetness variables. This would allow for more efficient, cost-effective, and environmentally friendly management of stem rust epidemics. This study also laid a foundation for advanced understanding of stem rust outbreaks in relation to more relevant climate-crop factors which need to be confirmed in various geographical locations in the future.

### 3.2 Non-race specific resistance

Non-race specific resistance is also known as quantitative resistance, adult plant resistance, slow rusting resistance, horizontal resistance, partial resistance, polygenic resistance, standard resistance, discipline resistance, or minor gene resistance. This type of resistance is referred to as quantitative resistance because it is primarily determined by the amount of disease symptom improvement, which can be quantified by different infection types like necrotic and chlorotic regions with constrained sporulation, reduced spore production per infection site, smaller uredinial size, as well as lesion size and area (Lagudah, 2011). Such resistance is often quantitative in nature, featuring a partial resistance phenotype in which the pathogen’s growth is inhibited but no evident immune response is shown. It is referred to as horizontal resistance because it is active against many different races of the pathogen. It is non-specific resistance because the resistance is not necessarily specific to a precise race for it to be most effective. It is referred to as minor gene resistance since a small number of genes control the resistance. When it comes to resistance expression, minor genes have limited impacts and display quantitative segregation, while major genes have substantial effects (Maurya et al., 2021). It is referred to as standard/partial/field resistance since it is the most effective partial and field tolerant resistance. However, even though those descriptions sound appealing, there are several exceptions to this broad group. This resistance manifests itself in wheat at later stages of development and is thus referred to as APR (Li et al., 2014).

After the successful cloning of many wheats adult plant rust resistance genes in recent years, researchers have gained some insights into the processes of non-race specific resistance. Pre-infection components may also play a role in a few quantitative resistance cases, while sporulation is not necessarily impacted in a few qualitative resistance cases. Accurate levels of race non-specific resistance need the participation of genes with effects ranging from minor to moderate in importance. Slow rusting is the term used to describe this trait, which is often related to adult plant resistance (Lagudah, 2011). Even when 4 or 5 genes are combined, most slow rusting genes do not provide a suitable degree of resistance, particularly under severe disease pressure, but the amount of resistance may reach up to near immunity (Singh et al., 2000). It was observed that the interaction or cumulative actions of known and undiscovered adult plant resistance genes contribute significantly to the improvement of resistance durability (Gupta and Saini, 1993). Using transgressive segregation, combining various genes, including slow rusting adult plant resistance genes, might increase the expression of resistance (Singh et al., 2013). *Yr36*, *Lr67/Yr46/Sr55*, and *Yr34/Yr18/Sr57* cause leaf-tip necrosis and rapid senescence, like the uncloned *Lr46/Yr29/Sr58* gene. These behavioral similarities suggest a single mechanism, consistent with the lack of additivity when these genes are coupled. Significantly, race-specific and race-non-specific resistance genes commonly demonstrate additivity, suggesting they should be employed together for improved protection (Ellis et al., 2014).

Several genes are involved in quantitative disease resistance, with each gene contributing to a different level of resistance. Since quantitative disease resistance reduces the selection pressure against pathogen variants, those that overcome a single quantitative resistance locus have no advantage over their counterparts in survival and reproduction. Consequently, quantitative disease resistance is more likely to be long-lasting than *R* gene-mediated resistance (Parlevliet, 2002). Quantitative resistance, in contrast to qualitative resistance, exhibits strikingly different properties. Unlike the majority of NLR-encoding *R* genes, certain adult plant resistance genes showed high durability in the field, such as *Sr2* (Ellis et al., 2014), and *Lr34/Yr18/Sr57* (Krattinger et al., 2009; Risk et al., 2013), which have been very effective in the field against many races of stem rust and leaf rust, respectively, in a variety of environments for almost 100 years. Combining *Lr34* with other adult plant resistance genes namely *Lr46, Lr67* and *Lr68*, significantly reduced damage from leaf rust (Silva et al., 2015). The determination of their genetic nature through cloning is important to predict their durability. Most of the identified wheat rust resistance genes are race specific all-stage resistance, and only a few are adult plant resistance genes such as *Lr34/Yr18/Sr57, Lr46/Yr29/Sr58, Lr67/Yr46/Sr55, Lr68, Sr2, Sr13*, *Sr21*, *Yr30*, *Yr36*. Among the adult plant resistance genes, *Lr34/Yr18/Pm38/Sr57*  (Singh et al., 2012)*, Lr46/Yr29/Pm39/Sr58*  (Singh et al., 2013b), and *Lr67/Yr46/Pm46/Sr55*  (Herrera-Foessel et al., 2014) confer pleiotropic adult plant resistance to all three rust pathogens plus powdery mildew caused by the fungal pathogen *Blumeria graminis* f. sp. *tritici* (William et al., 2003; Lillemo et al., 2008).

Wall-associated kinases (WAKs) represent a diverse cell surface immune receptor sub-family, specific to plants. WAKs confer resistance *via* different mechanisms ranging from non-specific quantitative resistance to a high level of specific resistance against particular races of pathogens (Kou and Wang, 2010). Wheat TaWAK6 a non-arginine-aspartate wall-associated kinase, with an extracellular GUB domain, a calcium-binding epidermal growth factor domain, and a cytoplasmic serine/threonine kinase domain was shown to be important for the development of quantitative and adult plant resistance (Dmochowska-Boguta et al., 2020). When plants reach the adult stage and the weather warms, high-temperature adult plant resistance increases or rises, while high-temperature seedling plant resistance is induced following exposure of wheat seedling to a temperature of 20°C for just 24 hours during the early stage of Pst incubation (Wang et al., 2017a; Wang et al., 2019b). TaRPM1, an NBS-LRR gene in wheat, has been shown to favorably control high-temperature seedling plant resistance to Pst *via* the salicylic acid signaling pathway (Wang et al., 2020c). Several genes are involved in quantitative disease resistance, with each gene contributing to a different level of resistance. Since quantitative disease resistance lessens the selection pressure against pathogen variations, this increases breakdown risks and there is need to incorporate other influential disease management tools in particular proper planting dates for each region and defense system induction by natural products such as silicon. Silicon is a bioactive element that has been shown to effectively alleviate biotic and biotic stresses, and enhance resistance against pathogenic fungi (Wang et al., 2017c). Silicon-induced biochemical or molecular resistance during plant-pathogen interactions was dominated as joint resistance, involving activation of defense-related enzymes, regulation of the complex network of signal pathways, stimulation of antimicrobial compound production, and activation of the expression of defense-related genes (Wang et al., 2017c). Understanding plant-microbe interactions mediated by silicon will aid in the efficient usage of this bioactive element to increase crop yield and improve plant disease resistance. Because many plants are unable to accumulate silicon at sufficient levels to be useful, genetically modifying the root’s silicon absorption capacity may help plants accumulate more silicon and, as a result, increase their ability to withstand biotic and abiotic challenges (Ma and Yamaji, 2006). Recently, concurrently occurring of leaf rust infection and drought stress was alleviated by exogenous silicon and hydrogen sulfide (Naz et al., 2021). The expression of stress and pathogenesis-related proteins will be studied in the future under individual and interactive stress conditions. Although numerous studies have shed light on the physical, biochemical, and molecular levels of silicon-mediated resistance, detailed mechanisms of silicon-mediated plant-microbe interactions, such as plant signaling transduction and transcriptome regulation of defense-related pathways, require further investigation (Wang et al., 2017c). Apart from our limited knowledge about the structural and functional characteristics of silicon transport proteins, there are many unanswered questions, such as the role of silicon in interactions with signaling molecules under normal and stress conditions, its impact on nutrient uptake, its influence on the photosynthetic machinery, and its role in phytohormones integration. Gaining a deeper understanding of silicon biology may be beneficial to a variety of disciplines such as agriculture, ecology, and industrial applications. Although significant progress has been made in the development of methods for the control of wheat stripe rust, research on the interplay between climate-disease-planting-date-resistance interaction in this pathosystem merits further investigation. Naseri and Marefat (2019) reported the first study that integrated the effects of relative humidity and air temperature, disease onset, maturity date, planting date, and wheat resistance on the severity of stripe rust. Because of the relationship between environment, maturity, and planting date, we now have a better knowledge of cultivar resistance in a rust-wheat pathosystem. Therefore, these findings provide a basis for using climatic conditions, maturity, and planting date as influential traits for selecting wheat varieties to improve disease prediction accuracy, resistance durability, and management efficacy (Naseri and Marefat, 2019). Further study is needed to assess the relevance of current linkages to diverse geographical locations, pathogen and host genotypes, and environmental variables.

## 4 Cloning and characterization of wheat R genes

Over the past century, the wheat-rust pathosystem was researched intensively among host–pathogen interactions because of its significance to the economy. Wheat rust resistance genes started to be isolated and cloned in the late 1990s, and the first rust resistance gene to be successfully isolated and characterized was reported in 2003 (Feuillet et al., 2003). The allele of *Lr34/Yr18/Sr57* which in wheat confers durable resistance against rust pathogens, was cloned in 2009 (Krattinger et al., 2009). 32 rust resistance genes have been cloned so far using a variety of strategies, with more than half of these having been cloned only in the last 5 years (Zhang et al., 2020b; Hafeez et al., 2021). This was made possible by the availability of the world’s first high-quality reference genome for wheat (Chinese Spring RefSeq v1.0) and the development of various approaches for reducing genome complexity in order to allow targeted resequencing analyses to be conducted. Target-sequence enrichment and sequencing (TEnSeq) pipelines, in particular, were utilized for the discovery of 16 out of the 32 genes. TEnSeq pipelines have several approaches that include mutagenesis and the resistance gene enrichment and sequencing (MutRenSeq) (Steuernagel et al., 2016; Marchal et al., 2018; Zhang et al., 2021a; Yan et al., 2021; Hewitt et al., 2021), association genetics with resistance gene enrichment sequencing (AgRenSeq) (Arora et al., 2019; Yu et al., 2022), mutagenesis chromosome flow sorting and short-read sequencing (MutChromSeq) (Sánchez-Martín et al., 2016), targeted chromosome-based cloning (TACCA) (Thind et al., 2017), mutagenesis isoform sequencing and transcriptome deep sequencing (MutIsoSeq), and bulked segregant RNA-Seq (BSR-Seq) (Lin et al., 2022). TACCA and MutChromSeq are based on the purification of individual chromosomes from wheat lines, whereas MutRenSeq and AgRenSeq are based on NLR-targeted DNA capture by hybridization (Zhang et al., 2020b). MutIsoSeq integrates isoform sequencing (Iso-seq) and transcriptome deep sequencing (RNA-seq) (Wang et al., 2022).

Three major *R* gene families in wheat have been reported based on their sensitivity, specificity, and durability (Krattinger et al., 2016). The families include genes that provide resistance to a single pathogen race only (NLR family), genes that confer non-race specific resistance to multiple races of multiple pathogens concurrently (ABC family); and genes that confer non-race specific resistance to all races of a single pathogen species (START family proteins). The most prevalent type of proteins encoded by plant *R* genes are nucleotide-binding site leucine-rich repeat (NBS-LRR) proteins, which act primarily by recognizing the effector molecules secreted by pathogens to suppress host defense responses (Jones et al., 2016). Plant NLR gene families have radiated and diversified to aid in the battle against potentially infectious pathogens, for example, through localized gene duplication or mutation within their LRR domains that bind pathogen effectors (Sarris et al., 2016). Furthermore, certain NBS-LRRs have additional integrated domains, the most common of which are kinase and DNA-binding domains (Andersen et al., 2020; Steuernagel et al., 2020), which are thought to be important in receptor activation or downstream signaling (Sarris et al., 2016). RAR1 and SGT1 are molecular chaperones needed for *R* gene expression, such as *Lr21* (Huang et al., 2003) and *Lr24* (Zhang et al., 2011).

**Figure 1:**
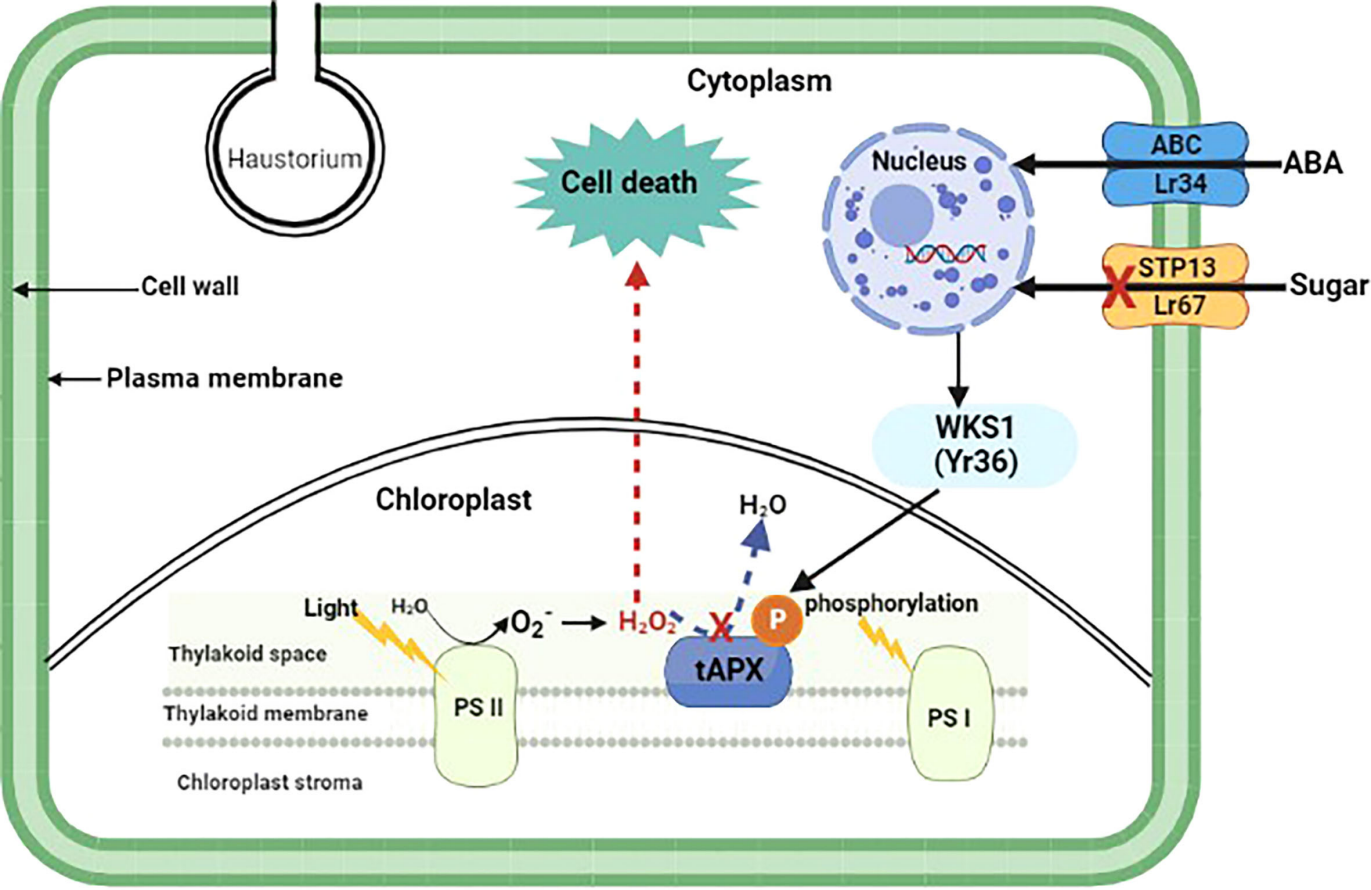
Proposed model of adult plant resistance gene function. Pathogen-specific (*Yr36*) and multi-pathogen (*Lr34* and *Lr67*) adult plant resistance genes and their involvement in plant cell signaling and defense pathways. Yr36, a wheat kinase START1 (WKS1) protein, mediates resistance to wheat stripe rust through phosphorylation of photosystem II manganese-stabilizing polypeptide protein complex (PsbO) found in the chloroplast thylakoid membrane. Phosphorylated PsbO is rapidly degraded by proteases, yielding PsbO-free PSII, which has a lower photosynthesis rate and serves as a source of ${\text{O}}_{2}^{-}$ , which is then converted to H_2_O_2_. Due to WKS1-mediated phosphorylation, H_2_O_2_ cannot be effectively destroyed by thylakoid ascorbate peroxidase (tAPX) and it accumulates, inducing cell death which curbs *Pst* growth. Lr34 and Lr67 are adenosine triphosphate-binding cassette (ABC) and sugar transporter (STP) proteins that confer multi-pathogen resistance through regulation of abscisic acid (ABA) and hexose sugar molecules, respectively.

25 out of 32 cloned wheat *R* genes that give all-stage resistance encode NBS-LRRs (**Table 1**). Furthermore, all except two of these 25 NBS-LRRs have CC domains near their N-termini; the exceptions are *Yr7* and the allelic *R* genes *Yr5/YrSP*, which each have an N-terminus integrated BED zinc finger domain (Marchal et al., 2018). *Sr60*, is race-specific but confers a partial resistance phenotype and encodes a protein with two putative kinase domains (Chen et al., 2020). *YrU1* encodes a protein with N-terminal ANK repeat and C-terminal WRKY domains (Wang et al., 2020b). The activity of *YrU1* in wheat’s resistance to stripe rust is dependent upon the homo-dimerization of CC and ANK repeats (Wang et al., 2020b). *Lr14a* encodes a protein with an N-terminal domain containing 12 ANK repeats followed by six predicted transmembrane helices (Kolodziej et al., 2021). There is an increasing interest in more long-lasting sources of resistance due to continuing alterations and swift spread of *Pst* populations worldwide. Four adult plant *Yr* genes have so far been cloned. *Yr36* encodes a protein with a kinase and START lipid-binding domain, WHEAT KINASE START 1 (WKS1) (Fu et al., 2009) WKS1 mediates resistance by phosphorylating the photosystem II manganese-stabilizing polypeptide (PsbO) protein complex found in the chloroplast’s thylakoid membrane (**Figure 1**). Phosphorylation of PsbO results in the production of reactive oxygen species and ultimately H_2_O_2_, which induces cell-death-mediated defense against the stripe rust fungus. WKS1 then phosphorylates the enzyme thylakoid ascorbate peroxidase (tAPX) to inhibit H_2_O_2_ degradation  (Gou et al., 2015; Wang et al., 2019d). Interestingly, *Yr36* showed additive resistance with both *Lr34* and *Lr67* suggesting different mode of action to these other two genes. *Hordeum vulgare* (*Hv*)*STP13*, like the pathogen-susceptible version of wheat *Lr67*, encodes a protein involved in the transport of glucose molecules. Because pathogens are sensitive to changes in these sugar transporter functions, powdery mildew-resistant barley lines were recently developed by mutating *HvSTP13* (Skoppek et al., 2022). Following that, it was discovered that introducing wheat *Lr67* into barley disrupted the functioning of *HvSTP13*, since the resulting transgenic lines were resistant to barley leaf rust and powdery mildew diseases. This resistant form of *HvSTP13*, like *Lr34* in barley, is produced early in plant development in transgenic barley lines, and its protein product triggers pathogenesis-related genes to induce defense (Dinglasan et al., 2022). Nevertheless, *Lr67-*mediated multi-pathogen resistance is conferred by a sugar transporter protein (STP) which belongs to the sub-group STP13 (**Figure 1**).

However, not all adult plant resistance genes confer broad-spectrum resistance. Notably, wheat tandem kinase 1 (WTK1), expressed by the gene *Yr15*, and wheat tandem kinase 3, which is encoded by the gene *Pm24*, both confer broad spectrum all-stage resistance against more than three thousand genetically varied *Pst* isolates and thirty-six tested isolates of *Bgt*, respectively (Klymiuk et al., 2018; Lu et al., 2020). *Yr15* has recently been found to be allelic with *YrG303/YrH52* (Klymiuk et al., 2019). In recent years, a hitherto unknown category of intracellular receptors known as tandem kinase proteins (TKPs) has emerged as an example of an unusual class of resistance proteins. TKPs consists of two distinct kinase domains that are linked together by a linker region (Klymiuk et al., 2021). Wheat and its relatives have been responsible for the discovery of five out of a total of six TKPs that have been functionally characterized in plants (Klymiuk et al., 2018; Chen et al., 2020; Lu et al., 2020; Gaurav et al., 2022). *Sr62* is a tandem kinase gene that was recently discovered to confer significant levels of resistance against twelve geographically diverse *Pgt* isolates in wheat (Yu et al., 2022). According to mutation study results, both of the kinase domains of *Sr62* were necessary for resistance (Yu et al., 2022). There is currently a lack of understanding about the molecular mechanism behind TKP-mediated resistance. It has been suggested that the pseudokinase domain, or one of the kinase domains, might serve as a decoy for the detection of pathogen effectors, and the kinase domain once activated, could begin downstream defense signaling (Klymiuk et al., 2021). Recently, a MutIsoSeq study unraveled the evolving function of kinase fusion proteins in wheat rust resistance. *Lr9* and *Lr58* were found to have the same coding sequence and cytogenic and haplotype analyses revealed that they originated from an identical translocation event (Wang et al., 2022). Both genes encode an unusual tandem kinase fusion protein with an N-terminal tandem kinase domain followed by a von Willebrand factor A (vWA) domain and a Vwaint domain in the C-terminus. Furthermore, the two genes both confer strong broad-spectrum resistance against many *Pt* races under regulated conditions (Wang et al., 2022). A comprehensive study on the hexaploid wheat genome identified NLR, ABC, and START genes, as well as how they are physically linked to *R* genes at both seedling and adult stages (Peng and Yang, 2017). According to the findings, the ABC and START genes are more likely to be co-located with non-race specific adult resistance genes, while the NLR genes are more likely to be co-located with race-specific resistance genes that are often expressed at the seedling stage (Peng and Yang, 2017). Given the tendency of *NLR* genes to be overcome by pathogen mutation to virulence it seems unlikely that this latter type of adult plant resistance will remain durable. Resistance genes that have been cloned might be potentially beneficial in the assembly of transgenic multigene cassettes for the purpose of producing robust and long-lasting resistant cultivars to battle rapidly emerging virulent fungal pathogens (Wulff and Moscou, 2014; Luo et al., 2021).

## 5 Molecular basis of interactions between wheat and rusts

Plants have a complex innate immune system that helps them to fight different pathogens. Wheat defense responses against pathogens consist of a strongly regulated and multifaceted molecular network which involves an extensive gene expression reprogramming during pathogen infections (Waheed et al., 2021). Distinct plant immune receptors recognize pathogen-derived chemicals, inducing different defensive responses that converge into common signaling pathways (Lu and Tsuda, 2020). PRRs recognize PAMPs/MAMPs, and activate PTI to induce defensive mechanisms against non-adapted infections. PAMPs recognition cause plant signals such as an oxidative burst, calcium influx, activation of the mitogen-activated protein kinase (MAPK) cascades, nitric oxide burst, ethylene synthesis, callose deposition at the cell wall, and expression of defense-related genes implicated in immune responses (Boller and Felix, 2009). Calcium signals affect salicylic-mediated plant immunity. Calcium acts as a secondary messenger in intra- and extracellular communication, including signal transfer (DeFalco et al., 2009). Because of its cytotoxicity, cytosolic Ca^2+^ levels in living cells must be kept low (approximately 10^˗8^ to 10^˗7^ M), so Ca^2+^ is sequestered in intracellular stores or the apoplast *via* active transport, generating enormous electrochemical potential gradients across membranes (Clapham, 2007; Edel et al., 2017; Costa et al., 2018). Calmodulins (CaMs), calcium-dependent protein kinases (CDPKs), and calcineurin B-like proteins (CBLs) sense and decode transient Ca^2+^ changes. CIPKs are necessary for biotic stress tolerance when plants interact with pathogens (Liu et al., 2019). CaM protein was shown to be involved in the early stages of signal transduction pathway during wheat-*Pt* interactions. Preceding findings revealed that TaCaMs were involved in early stages of incompatible interaction processes and play a critical role in the wheat resistance signal transduction pathway against *Pt*. *TaCAMTA4*, a putative calmodulin-binding transcription activator, was found to function as a negative regulator of wheat defense response to *Pt* (Wang et al., 2019e). Calcineurin B-like interacting protein kinases (CIPKs) are critical for the plant’s tolerance to biotic stresses during plant-pathogen interactions (Liu et al., 2019). *TaCIPK10* was demonstrated to positively regulate wheat defense responses to *Pst* by acting as molecular bridges between Ca^2+^ and downstream defense components. Regulation of wheat resistance to *Pst* was also found to be enhanced by the interaction and phosphorylation of *TaCIPK10* with TANH2 (Liu et al., 2019).

Wheat resistance to *Pt* was induced by extracellular Ca^2+^ influx, which is regulated by the calcium signaling system (Liu et al., 2015). Ca^2+^ mediates *Pt*’s incompatible hypersensitive response mechanism (Hou et al., 2007; Liu et al., 2010b; Qiao et al., 2015). *TaCRK2* expression was upregulated after *Pt* infection and was highly inhibited by ethylene glycol tetraacetic acid, a chelating agent, in incompatible interactions between wheat and *Pt*, while it was not expressed in compatible interactions. This led Liu and colleagues to hypothesize that it could be related to Ca^2+^ signaling and resistance to *Pt* (Liu et al., 2019). In a recent study, it was also discovered that *TaCRK2* gene is controlled by a Ca^2+^ signal and positively regulates leaf rust resistance in wheat (Gu et al., 2020). It can be concluded that, during wheat-rust pathogens interactions, the complex molecular network of wheat cells undergoes extensive transcriptional reprogramming to activate a cascade of plant defense responses to combat infections.

### 5.1 The network of resistance to different rust races

The network of resistance can be analyzed using RNA-seq and metabolomics. Six *Pt* races were inoculated onto a susceptible wheat variety, and samples were collected six days later, shortly before pustule eruption (Neugebauer et al., 2018). A time course study was utilized to analyze the expression pattern of 63 wheat genes during infection over the first seven days after inoculation. Differential expression of 47 wheat genes was confirmed, with two genes being linked to race-specific gene expression, indicating that variation in *Pt* effector repertoires resulted in distinct wheat interactions. Races from two separate *Pt* lineages were linked to differential expression of an endoplasmic reticulum molecular chaperone gene. In addition, differential expression of an alanine glyoxylate aminotransferase gene was shown in *Pt* races with virulence changes for leaf rust resistance (Neugebauer et al., 2018). cDNA AFLPs were used to identify transcript-derived fragments that were differentially expressed during the first week of *Pst* infection (Wang et al., 2009). A downregulation of the expression of chlorophyll a-b binding proteins and RuBisCO, and an upregulation of the expression of ten transcript-derived fragments associated with signal transduction functions was found during the early stages of infection (Wang et al., 2009). Seventy-three transcripts were induced by *Pst* infection in a compatible interaction and accumulation of transcripts peaked at 24 hpi (Coram et al., 2010). Of the transcripts discovered, 25 were related to defense, six to signal transduction, seven to protein and carbohydrate transport, eight to metabolism, 19 to biotrophic interactions, four associated with electron transport, and 25 had unidentified functions (Coram et al., 2010). Furthermore, 42 probe sets were found to be upregulated and one probe set was found to be repressed in a *Pst*-wheat compatible interaction (Bozkurt et al., 2010). The bulk of the probe sets were involved in defense responses, whereas nine of them were associated with glucose metabolism (Bozkurt et al., 2010).

Wheat-*Pt* incompatible interactions triggered peroxidases and NADPH oxidases, dubbed respiratory burst oxidase homologs (Rboh), and reactive oxygen species (ROS) accumulation in stomata and mesophyll cells around the infection site (Orczyk et al., 2010). Rboh proteins are directly controlled by calcium ions through N-terminal EF-hand calcium-binding motifs (Sagi and Fluhr, 2006). Before pathogen-induced defense reactions, cytosolic Ca^2+^ spikes trigger Rboh activity and an oxidative burst (Zhao et al., 2005). The expression of the Rboh-like expressing gene (JG968934) closely matches oxidative burst in both Thatcher and *TcLr9* lines (Orczyk et al., 2010; Dmochowska-Boguta et al., 2013). Incompatible interaction reactions increased the transcription of WAKs, which may function as signal transducers due to their transmembrane localization, calcium-mediated signaling, and Rboh-like proteins, which may have roles in oxidative burst and micronecrotic processes (Dmochowska-Boguta et al., 2013). Altogether, these findings showed the involvement of NADPH oxidases and peroxidases in wheat defense against pathogen infections. *De novo* transcriptome assemblies discovered differentially expressed genes during wheat-*Pt* compatible interaction. There was an upregulation of the expression of glutathione-transferase genes and reactive oxygen species enzymes, resulting in oxidation state reduction in susceptible cultivars compared to resistant ones (Chandra et al., 2016). Fifty-nine putative rust-induced RNAs were found in the flag leaves of Thatcher-*Lr34/Yr18/Sr57* spring wheat isogenic line (Hulbert et al., 2007). Furthermore, 102 and 113 rust response wheat genes linked to the *Yr5* and *Yr39* genes, respectively were identified (Chen et al., 2013). When infected with stripe rust or powdery mildew, comparative transcriptomics revealed distinct changes in the defense response genes (Zhang et al., 2014; Hao et al., 2016). In a stripe rust adult plant resistance study, an upregulation of pathways involved in systemic symptom development in response to *Pst* infection in adult wheat plants was reported (Hao et al., 2016). However, in seedling wheat-stripe rust reaction, qualitative resistance was established, where a major *Yr* gene was influencing the energy-related, defense-related, signal transduction, transcription regulation and metabolism related pathways (Wang et al., 2010). Similarly, a study on *Lr10*-mediated wheat leaf rust interaction revealed specific gene sets contributing to cell wall fortification, signaling, peroxide oxidation and energy metabolism (Manickavelu et al., 2010). However, plant survival is ensured by excessive ROS detoxification *via* MAE and oxidase genes due to the presence of the *Lr28* gene which mediates effector recognition and induces a strong hypersensitive response through upregulation of candidate *MSC, CK, RBOH* and terpene synthase genes.

Transcriptome profiling and quantification of differential expression of genes and proteins is essential in elucidating regulatory pathways and gene-networks due to their broad transcript coverage, high sensitivity, and allele-specific differential expression, (Lindlöf et al., 2015; Chawade et al., 2016; Chawade et al., 2018). RNA-seq analysis of wheat seedling leaves infected with *Pst* identified 520, 148, and 1439 differentially expressed genes that were either transiently upregulated or downregulated at 1-, 3-, and 7-days post inoculation, respectively. Gene ontology and Kyoto Encyclopedia of Genes and Genomes enrichment analysis revealed the involvement of various biological processes including MAPK signaling pathway, oxidative phosphorylation, flavonoid biosynthesis, phenylalanine metabolism, and photosynthesis, in wheat cultivar SM126’s response to *Pst* infection (Wang et al., 2021b). Four genes were differently expressed in SM126’s response to *Pst* infection at the three time periods. Two of them (*TraesCS3B02G192400* and *TraesCS5B02G018700*) were previously shown to be involved in the metabolism of zinc (Bhatta et al., 2018) and nitrogen (Karim et al., 2020). Zinc affects plant-pathogen interactions through its crucial function in the activation/stabilization of metalloenzymes (Fones et al., 2010; Cabot et al., 2019). Nitrogen contributes to plant defense responses through the control of plant primary metabolism during plant-pathogen interactions (Wang et al., 2019c). Therefore, it was postulated that these genes may play essential roles in the unique resistance networks of SM126. This study also revealed the involvement of various differentially expressed genes in PTI and ETI pathways (Wang et al., 2021b).

Cysteine-rich receptor-like kinases (CRKs) are involved in transduction pathways upon pathogen perception. A wheat CRK gene (*TaCRK10*) from wheat variety Xiaoyan 6 (XY6) carries high temperature seedling plant resistance to *Pst* stripe rust. *TaCRK10* serves as an important sensor of *Pst* infection and high temperatures and activates wheat resistance by regulating nuclear processes. These findings paved a way for the elucidation of molecular mechanisms of wheat high temperature seedling plant resistance to *Pst* and promoted efforts in developing wheat varieties with resistance to stripe rust (Wang et al., 2021a). When wheat plants reach the adult stage, high temperature adult plant resistance expresses or rises when the weather warms, while high temperature seedling plant resistance expresses when wheat seedlings are momentarily exposed to 20°C for just 24 hours during the early stage of *Pst* incubation (Wang et al., 2017a; Wang et al., 2019b). *TaRPM1*, an NBS-LRR gene in wheat, was shown to positively regulate high temperature seedling plant resistance to *Pst via* salicylic acid-signaling pathway (Wang et al., 2020c). RNA-seq analysis established an upregulation of *TuRLK1* transcript level after inoculation with *Pst* in the presence of YrU1 in *Triticum urartu* accession PI428309. Silencing of *TuRLK1* severely compromised the resistance of *YrU1* to *Pst* CY33. This study demonstrated the importance of *TuRLK1* in immune response mediated by the unique NLR protein YrU1, and *TuRLK1* might play an important role in disease resistance to other pathogens (Zou et al., 2022). Therefore, YrU1 likely functions as a typical NLR protein that elicits effective ETI after recognition of the cognate effector proteins derived from biotrophic pathogen *Pst*. How YrU1 activates plant immunity, and whether PRRs/co-receptors or other key components of PTI are required for YrU1-mediated plant immunity remains to be determined. This study clearly showed that an RLK, a key PTI component, is indispensable for ETI in fungal disease resistance, which is consistent with the previous studies on bacterial disease resistance (Zou et al., 2022). Chaperones confer plant resistance by maintaining cell homeostasis during infection. Heat shock proteins (Hsp) Hsp60, Hsp70, and Hsp90 have been identified as pathogenesis-related (PR) proteins. Wheat resistance to *Pgt* provided by *TaRLK-R* and wheat resistance to *Pt* conferred by *Lr21* both rely on Hsp90s (Scofield et al., 2005; Botër et al., 2007).

### 5.2 Resistance related genes

#### 5.2.1 PR proteins for resistance against rusts

The expression of a collection of genes, including *PR* genes, is specifically associated with the manifestation of systemic acquired resistance (SAR) in plant defense responses against pathogen infection. The induction of PR proteins by different pathogens in many plants has been reported, and are thought to function as a key component of the SAR machinery within signaling pathways (Ward et al., 1991; Van Loon and Van Strien, 1999; Prasad et al., 2020). Further defense response mechanisms are elicited by the enzymatic products of PR proteins (Fritig et al., 1998). To date, well known and documented inducible PR proteins (PR1-PR17) consist of 17 families (Sels et al., 2008), and the most prevalent are PR1, PR2, and PR5, which accumulate both locally and systemically, implying that they are involved in SAR (van Loon et al., 2006). Several studies have also shown that PR1, PR2, and PR4 are essential in enhancing wheat leaf rust resistance (Gao et al., 2015; Casassola et al., 2015; Zhang et al., 2017a; Prasad et al., 2019). Accumulating evidence from genetic and biochemical studies showed that pathogen invasive growth and proliferation is inhibited by the binding of PR1 to sterols (Gamir et al., 2017). *PR1* is a conserved gene that encodes an enzyme called β-1,3 glucanase which is essential for breaking down cells wall of fungal pathogens and hydrolysis of cell wall glucans. β-1,3-glucanases and chitinases are the most two studied classes of PR proteins in pathogen-host interaction studies (Prasad et al., 2020). Some wheat-*Pt* interaction studies reported the existence of a synergistic function between β-1,3-glucanase and chitinase (Anguelova-Merhar et al., 2001; Gupta et al., 2013). Consequently, the secretion and accumulation of β-1,3- glucanase and chitinase in the apoplastic space upon fungal infection highly contributes to plant defense against pathogen invasion (Kauffmann et al., 1987). Liu and colleagues reported an upregulation of *TaGlu*, a wheat β-1,3-glucanase gene in both compatible and incompatible wheat-*Pst* interactions, but no transcript change occurred during the first 12 hours in both interactions (Liu et al., 2010a). Similarly, *TcLr19Glu* isolated from near isogenic wheat line TcLr19, was induced by *Pt* infection. The expression of *TcLr19Glu* in incompatible interaction appeared earlier than that in the compatible interaction and the accumulation of transcripts was much higher than in the compatible interaction at different time points. This showed that *TcLr19Glu* is involved in wheat resistance against *Pt* (Gao et al., 2016). Nonexpressor of pathogenesis-related genes 1 (NPR1) was discovered to be a crucial transcriptional regulator in defense responses of various plants against pathogen infections. Although nine NPR1 homologues (*TaNPR1*) were identified in wheat, little is yet known about the functions of the *NPR1*-like genes in wheat defense response against rust pathogens. Downregulation of all the *TaNPR1* homologues by virus-induced gene co-silencing led to increased resistance to stem rust (Wang et al., 2020d). Wang and colleagues proposed a novel mechanism of NPR1 activity in wheat at the *Ta7ANPR1* locus, *via* a NB-ARC–NPR1 fusion protein, which negatively regulates the resistance against stem rust infection (Wang et al., 2020d).

*PR2* proteins can also be classified as β-1,3-glucanases with β-1.3-endoglucanase functions in their structure (Lata et al., 2022). They were reported to be responsible for weakening fungal cell wall by catalyzing the hydrolytic cleavage of 1,3-β-D-glucosidic linkages found in β-1,3-glucans (Singh et al., 2014). Throughout the course of wheat-*Pst* interaction, these enzymes displayed consistent expression at every time point (Lata et al., 2022), and they restricted pathogen invasive growth and proliferation by inhibiting formation of haustorial mother cells and secondary infection transmission. This clearly demonstrated the involvement of *PR2* proteins in wheat defense responses against pathogen infection. *PR4* genes encode endochitinase enzymes which are responsible for the breakdown of fungal cell wall chitins (Lata et al., 2022). During the early infection phases, *PR4* expression was increased in response to incompatible interaction (Lata et al., 2022). In many agricultural plants as well as model plants, the PR4 proteins are considered to be the signature genes that are involved in the jasmonic acid pathway (Ali et al., 2018). Thaumatin-like proteins (TLPs) commonly known as the PR5 family proteins are strongly induced by various abiotic and biotic stresses and they confer resistance in different plant species (Fierens et al., 2009; Petre et al., 2011). A PR5 protein, also known as *TaLr35PR5*, was discovered to be important in leaf rust resistance (Li et al., 2015). *TaLr35PR5* expression was upregulated during the early stages of wheat-*Pt* incompatible interaction, and this was linked to *Lr35*-mediated resistance in wheat (Zhang et al., 2018a). It was also established that, leaf rust resistance is linked to peroxidases (PR9) which is involved in different physiological functions such as plant defense (War et al., 2012). Peroxidases help to maintain host resistance by synthesizing structural barriers or by producing ROS and reactive nitrogen species that inhibit pathogen proliferation. Some peroxidases are involved in the reduction of H_2_O_2_, which may contribute to the susceptibility of wheat to leaf rust and powdery mildew (Savadi et al., 2018; Prasad et al., 2019). The PR9 proteins played a crucial role in the production of ROS, the development of mechanical barriers to prevent the pathogen from spreading, and the death of the pathogens during wheat-*Pst* interactions (Lata et al., 2022). The limited development and eventual death of the pathogen was associated with a higher expression of PR9 under incompatible interactions. The expression of *PRA2*, a Class III peroxidases family gene, was observed to be considerably higher under wheat-*Pst* incompatible interaction from 6 hpi, which might aid plant cells in mediating host resistance. Furthermore, it may limit pathogen invasion before the development of haustoria and limit pathogen dissemination through ROS-mediated resistance (Lata et al., 2022).

Many phenyl-propanoid pathways, including those involving lignin, flavonoid, and phenyl propanoid production, have been shown to play a key role in plant defense. There are eleven enzymes involved in these pathways, with the most important being phenylalanine ammonia-lyase (PR10) (Li et al., 2014). *PR10* genes display ribonuclease activity (Casassola et al., 2015). It was shown that enhanced *PR10* expression is associated with increased wheat resistance against *Pt* (Casassola et al., 2015; Prasad et al., 2019). *PR10* expression was enhanced exponentially during early infection stages in wheat-*Pst* compatible interactions; however, it was unable to maintain its level of expression and did not inhibit the development of the fungal pathogen (Lata et al., 2022). Type 1 non-specific lipid transfer protein precursor (LTP; PR14) is involved in plant defense through the deposition of extracellular cutin or wax which acts as a mechanical barrier to pathogen invasion (Serrano et al., 2014), or lipids required for membrane repair (Molina et al., 1993; Kader, 1996). After being infected with *Pt*, it was shown that the PR14 proteins, which represent a number of wheat LTPs, were upregulated in wheat cultivars harboring the *Lr34/Yr18/Sr57* genes (Hulbert et al., 2007). These LTPs bind to membrane lipids and transport them across membranes, making them plant innate immunity critical components. It was hypothesized that wheat *TaLTP3* plays an important role in defense response to rust infections since *Pst* effector PNPi targeted it (Bi et al., 2020). Wang and colleagues discovered that *TaPR1* interacts with TaTLP1 (TaPR5) in the apoplastic region, resulting in enhanced antifungal activity (Wang et al., 2020a). A considerable increase in resistance to both *Pst* and *Pt* was observed when a PR1 homology, *TaPR1a* was overexpressed in a transgenic wheat line (Bi et al., 2020). *TaLTP3* was suggested to be important in wheat resistance to *Pt* infection through the formation of a *TaLTP3-TaPR1a* complex in the apoplast, and this gives fresh insights into the functional roles of wheat PR proteins (Zhao et al., 2021). LTPs were discovered to be upregulated under wheat-*Pst* incompatible interaction during the time of membrane injury. This makes sense given that the transport of lipids is required for the healing of damaged tissue. On the other hand, during early stages of infection there was an upregulation of LTPs expression in compatible interaction (Lata et al., 2022). This suggests that some efforts are being made by the susceptible cultivar to defend itself from pathogen attack. However, at subsequent infection phases the pathogen was able to overcome such barriers that the host has constructed, leading to vulnerability (Lata et al., 2022).

#### Transporters in wheat resistance against rusts

Several studies have shed light on the function of membrane-localized transporter proteins in the resilience of plants to abiotic and biotic stresses (Krattinger et al., 2009; Krattinger et al., 2011; Moore et al., 2015). Membrane-localized transporter proteins are essential to the growth and development of plants. Pathogens directly target sugar transporters to get the carbohydrates necessary for their continued development and survival (Moore et al., 2015; Julius et al., 2017). ABC transporters are transmembrane proteins that utilize energy from hydrolysis of ATP for the transportation of substances across the cell membrane (Walter et al., 2015). The ABC transporters have two domains: a transmembrane domain and the nucleotide-binding domain. Plant ABC transporters serve vital functions in disease resistance and environmental interactions. Genome-wide transcription profiling of ABCG transporters in *Arabidopsis* revealed that 50% of these transporters are induced by jasmonic acid and salicylic acid (Osbourn, 1996; Kang et al., 2011). The *Lr34* gene encodes a full-size ABC transporter of the ABCG type, and its nucleotide-binding domain and C-terminal transmembrane domain comprise of a single polypeptide chain, organized as NBDTMD-NBD-TMD (Keller et al., 2012). The Lr34 ABC transporter was shown to be involved in plasma membrane remodeling characterized by intracellular phosphatidic acid accumulation and increased outward translocation of phosphatidylserine. In addition, the content of phosphatidylinositol 4,5-bisphosphate in the cytoplasmic leaflet of the plasma membrane was reduced in the presence of the ABC transporter (Deppe et al., 2018). The *Lr34res* allele is one of the most long-lasting sources of quantitative resistance in wheat (Krattinger et al., 2019). The encoded *LR34res* ABC transporter is essential in modifying the accumulation of 1-*O*-*p*-coumaroyl-3-*O*-feruloylglycerol, leading to increased accumulation of antifungal metabolites, essentially priming the wheat for defense (Rajagopalan et al., 2020). Metabolomics revealed the accumulation of phenylpropanoid diglyceride with an antifungal activity in *Lr34res* wheat cultivars which was later depleted upon rust infection. This emphasized a possible *Lr34res* role in mediating rust resistance by promoting higher accumulation of antifungal phenylproponoid metabolites (Rajagopalan et al., 2020).

The *Lr67* resistance allele was found to encode a protein that has lost its transport function, and this might as a result alter sugar balance between intracellular and extracellular leaf regions (Milne et al., 2019; McCallum and Hiebert, 2022). This may limit internal nutrients availability, demonstrating the importance of this gene is in defense responses (**Figure 1**). Alternatively, changing concentration of sugar within the apoplast may promote instigation of defense mechanisms (Dodds and Lagudah, 2016). Metallic phytosiderophores transportation necessitates yellow stripe-like (YSL) transporters which are similar to metal-nicotianamine complexes in structure. Recently, YSL transporters were found to be involved in pathogen-induced defense response (Islam et al., 2020). *TaYS1A* positively regulates wheat resistance to pathogen invasion by modulating the salicylic acid (SA) signaling pathway *via* ROS-dependent signals (Islam et al., 2020). During pathogen infection in plants, iron uptake and homeostasis might result in a burst of reactive oxygen species (Ryals et al., 1996; Ryals et al., 1997). Since *TaYS1A* transcription is stimulated by *TaNH2* by SA induction, it was postulated that its metal ion homeostasis role is responsible for reactive oxygen species accumulation that results in hypersensitive response in plant defense against pathogen infections (Islam et al., 2020). However, future studies need to verify this hypothesis.

### 5.3 The regulatory role of transcription factors in wheat resistance against rusts

**Figure 2:**
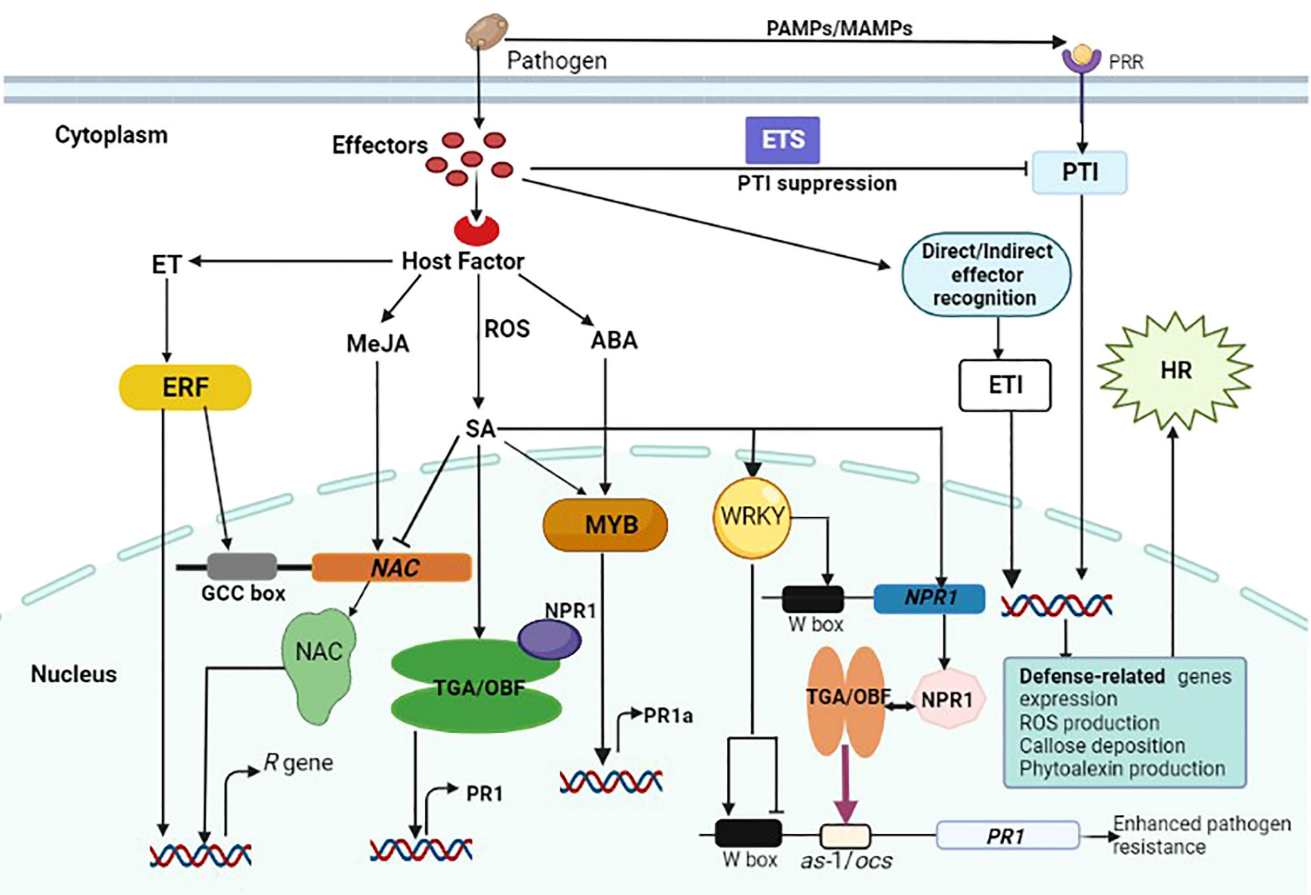
Schematic illustrations of a cross-talk between plant immune responses to fungal infection and the role transcription factors in gene expression regulation. Pathogen-derived conserved molecules (MAMPs) are recognized by pattern recognition receptors (PRRs), and this activates PTI. Pathogens induce susceptibility by interfering with the immune signaling network through effectors, resulting in effector-triggered susceptibility (ETS). Following direct or indirect effector recognition, plant R proteins activate host defense responses to stop pathogen growth, and this is regarded as effector-triggered immunity (ETI). Also, effector recognition triggers the induction of hormone signals and manipulated ERF, TGA, MYB and WRKY transcription factors to regulate the expression of *R* genes either directly or indirectly. Some transcription factors reciprocally regulate gene expression by binding to the corresponding promoter. There are many W-boxes within the NPR1 promoter that are necessary for gene expression. The NPR1 protein interacts with specific TGA/OBF proteins, which are bZIP transcription factors, to regulate the expression of the *PR1* gene. This increases their activity of DNA binding to the *as*-1/*ocs* element in the PR1 promoter (indicated by purple arrow) resulting in enhanced resistance to pathogen infection. In the PR1 promoter, WRKY proteins seem to exert both transcriptional activation and repression, hence their regulatory functions are complicated.

Efficiency of plant defense responses is enhanced by a wide range of transcription factors that are involved in downstream signaling cascades. They also orchestrate key processes involved in the growth and development of plants including transcription, post-transcription, translation and post-translation. Transcription factors have the ability to precisely bind to cis-acting regions in the promoter region of eukaryotic genes, thereby regulating the expression of many target genes (**Figure 2**) (Nakashima et al., 2009). In plants, transcriptional regulation of expression of stress-response genes is a critical component of their response to a variety of abiotic and biotic stresses (Singh et al., 2002). Transcription factors regulate differentially expressed gene products such as enzymes and dehydrins involved in reactive oxygen species elimination (Boller and He, 2009; Pieterse et al., 2009), which protect plant cells from infection (Jensen et al., 2007; Spitz and Furlong, 2012; Wang et al., 2019a; Liu et al., 2021). Transcription factors can be classified into families based on characteristics of their DNA-binding domains, which include WRKY, bZIP, MYB, NAC, ARF, bHLH, ERF/AP2, and MYC (Rushton and Somssich, 1998; Banerjee and Roychoudhury, 2015; Aslam et al., 2019; Falak et al., 2021). Although many studies have been focusing on the regulatory functions of transcription factors in plants, the role of transcription factors in biotic stress responses hasn’t been comprehensively reviewed, particularly in wheat. Therefore, this section will briefly discuss the regulatory role of WRKY, bZIP, MYB, and NAC transcription factors in wheat resistance pathways, emphasizing their functions in defense response to pathogen infection.

#### 5.3.1 WRKY transcription factors

WRKY is one of the best-studied classes of plant transcription factors involved in the regulation of a wide range of biological processes including development, physiology, and metabolism (Chen et al., 2017a). WRKY proteins are zinc-finger transcription factors that contain a DNA-binding domain and can bind to W-box repeats in defense-related gene promoters. The interaction between different WRKY transcription factors alter the activity of sequence-specific DNA binding, leading to varying degrees of plant defense responses (**Figure 2** **)** (Eulgem et al., 2000). The link between WRKY transcription factors and pathogen sensors is critical in the transmission of signals during host-pathogen interactions. WRKY transcription factors are nodes for a cross-talk across salicylic acid, jasmonic acid, and ethylene signaling pathways and they are involved in plant defense *via* these pathways (Li et al., 2006; Pandey and Somssich, 2009; Bakshi and Oelmüller, 2014). WRKY transcription factors were reported to be key components of plant innate immune system (Eulgem and Somssich, 2007). Wheat has more than 160 WRKY family members that has been identified (Okay et al., 2014; Satapathy et al., 2014). A few studies have so far reported the role of WRKY transcription factors in wheat resistance against rust pathogens. When HD2329 wheat cultivar was infected with a severe leaf rust fungus, *TaWRKY1B* expression was upregulated by 146-fold (Kumar et al., 2014a), demonstrating its involvement in wheat defense response to leaf rust pathogens. Degradome sequencing found an orthologue of TaWRKY2 in Xingzi 9104 that inhibits *Pst* CYR 32 (Feng et al., 2015). WRKY transcription factors can either positively or negatively regulate plant defense responses to pathogens. Recently, it was demonstrated that *TaWRKY70* positively regulates high temperature seedling plant resistance to *Pst* in wheat through salicylic acid- and ethylene -mediated signaling pathways (Wang et al., 2017a). Wheat *TaWRKY62* and *TaWRKY49* confer differential high temperature seedling plant resistance to *Pst.* It was established that *TaWRKY62* and *TaWRKY49* positively and negatively regulates wheat resistance to *Pst* respectively, by differential regulation of salicylic acid-, jasmonic acid-, ethylene-, and reactive oxygen species-mediated signaling (Wang et al., 2017b). *TaXa21* was speculated to be positioned upstream of the signaling pathway and responsible for perceiving environmental signals and transmitting them downstream such as to WRKY transcription factors. However, further research is needed to explore whether *TaXa21* in high temperature seedling plant resistance to *Pst* causes PTI or ETI responses (Wang et al., 2019b).

#### 5.3.2 bZIP transcription factors

In plants, basic leucine zipper (bZIP) regulates growth and responses to stress as a key transcription factor of the abscisic acid signaling pathway (Liang et al., 2022). bZIP transcription factors have two distinct motifs; a basic region for specific target DNA binding and a leucine zipper for transcription factor dimerization (Jakoby et al., 2002). Proteins containing bZIP domains bind to DNA sequences with an ACGT core (Hong et al., 2018). Genetic, molecular, and biochemical studies showed that bZIPs regulate a broad variety of plant functions, including pathogen defense (Thurow et al., 2005; Kaminaka et al., 2006). However, there is limited knowledge about the bZIP genes involved in defense responses in monocotyledonous plants, especially in wheat, because most studies have been focusing on the bZIP defense related genes of dicotyledonous plants like *Arabidopsis*, potato, tobacco and tomato. Therefore, identification and characterization of wheat bZIP genes involved in defense responses will help us understand disease resistance molecular mechanisms. Transcriptional analysis revealed that *Pst* stress promptly and significantly upregulated *TabZIP1* transcripts during the early stages of incompatible interaction. This suggested the involvement of *TabZIP1* in fungal-plant recognition and defense response against penetration (Zhang et al., 2008). Furthermore, *TabZIP1* transcripts were upregulated by exogenously applied methyl jasmonate (MeJA) and ethylene (ET). However, *TabZIP1* expression was not affected by salicylic acid treatment. This showed that the transcription factor protein encoded by *TabZIP1* gene may be involved in wheat defense response to *Pst* infection through the ethylene/methyl jasmonate-dependent signal transduction pathways (Zhang et al., 2008). bZIP transcription factors may either positively or negatively regulate plant defense responses to pathogens (Pontier et al., 2001; Singh et al., 2002). *TabZIP74* was found to positively regulate wheat stripe rust resistance and root development *via* mRNA splicing (Wang et al., 2019a). *Triticum aestivum* and *Triticum urartu* have 102 and 62 bZIP protein members, respectively (Jin et al., 2014).

#### 5.3.3 MYB TFs

Members of the myeloblastosis (MYB) gene superfamily contribute to plant growth and defense, garnering the interest of various global plant experts (Fujita et al., 2006; Wang et al., 2015b). MYB1R, R2R3, and MYB3R factors are subfamilies of MYB proteins with one, two, or three contiguous repetitions in the MYB domain. Plants have MYB-protein subfamily distinguished by the presence of the R2R3-type MYB domain (Hong et al., 2018). There are roughly 52 amino acid residues in total in the MYB domain, which adopts a helix-turn-helix conformation and intercalates into the primary groove of DNA (Dubos et al., 2010; Al-Attala et al., 2014). Members of the R2R3-MYB transcription factor superfamily were speculated to play a vital role in plant development, defense responses to abiotic and biotic stresses as well as enhanced disease resistance (Zhu et al., 2021). A member of the R2R3-MYB superfamily, *TaMYB29* have two highly conserved MYB domains and it regulates crosstalk across signaling pathways in wheat’s response to stripe rust. The expression of *TaMYB29* was significantly upregulated by *Pst* infection, salicylic acid, jasmonic acid, ethylene and abscisic acid treatment, indicating its involvement in wheat defense response to *Pst* (Zhu et al., 2021). Compared to non-silenced plants, knockdown of *TaMYB29* gene enhanced hyphal growth, substantially downregulated expression of pathogenesis-related genes and significantly reduced wheat resistance to *Pst* (Zhu et al., 2021). Therefore, these findings demonstrated that *TaMYB29* serves an indispensable role in wheat defense response to *Pst* through the regulation of crosstalk between multiple signaling pathways. Late elongated hypocotyl (LHY), a 1R-MYB transcription factor, is a plant essential gene that regulates the plant’s biological cycles. *TaLHY* gene encodes a protein with an MYB-DNA binding domain (Zhang et al., 2015). *Pst* influences the expression of *TaLHY* in wheat, similar to disease-resistance-related MYB TFs (Maeda et al., 2005). Zhang and colleagues demonstrated that *TaLHY* gene positively regulates wheat defense response against *Pst* infection (Zhang et al., 2015). *Pst* infection significantly upregulated *TaLHY* expression in wheat disease-resistant cultivars than in susceptible ones, indicating different effects of pathogenic fungal infestation on *TaLHY*. *Pst* infestation upregulated *TaLHY* expression was shown to be closely related to the salicylic acid-signal transduction pathway (Zhang et al., 2015). Given the intricacy of a plant’s systemic regulatory network, accurate gene regulation requires several transcription factors and signal molecules. *Pst* infection of SM126 leaves resulted in the establishment of robust and noticeable inductions in the MYB and WRKY transcription factors. Following *Pst* inoculation, the MYB and WRKY transcription factors were differentially expressed in SM126 leaves, indicating that the establishment of SM126 resistance to *Pst* necessitates the up- or down-regulation of transcription factors in order to regulate the plant defense signaling network (Wang et al., 2021b).

#### 5.3.4 NAC TFs

The regulation of cross-talk between various signaling pathways and transmission of pathogen-derived defense signals necessitates transcription factors to either suppress or activate downstream defense gene expression (Lorenzo et al., 2003; Anderson et al., 2004). NAC-type transcription factors (NAM, ATAF, and CUC) are involved in a wide range of plant biological regulating activities including developmental processes, plant growth, senescence, secondary cell wall synthesis, and biotic and abiotic stress responses (Nakashima et al., 2012; Puranik et al., 2012). Plant NAC proteins have a large family consisting of at least 151 members from rice and 117 members from *Arabidopsis* (Nuruzzaman et al., 2010; Xue et al., 2011). Several studies reported the involvement of certain NAC proteins in the regulation of plant defense responses through the activation of pathogenesis-related genes and hypersensitive response (**Figure 2**) (Jensen et al., 2007; Lin et al., 2007), whilst other few NAC genes may function as negative regulators of plant defense response through the inhibition of defense-related genes expression (Delessert et al., 2005; Le Hénanff et al., 2013; Wang et al., 2018). NAC transcription factors can also serve as virulence targets of pathogen effectors or as hypersensitive response and stomatal immunity modulators (Yuan et al., 2019). The expression patterns of NAC transcription factors are tissue-specific (Lin et al., 2007; Meng et al., 2009). *TaNAC4* expression was upregulated by *Pst* infection and also by methyl jasmonate, abscisic acid, abscisic acid, ethylene treatments. However, salicylic acid had no substantial effects on *TaNAC4* expression, indicating that *TaNAC4* gene serves as a key transcriptional activator in wheat defense responses to abiotic and biotic stresses (Xia et al., 2010a). TaNAC8 protein N-terminus and C-terminus contains a NAC domain and a transmembrane helices motif, respectively. Xia and colleagues demonstrated that the TaNAC8 C-terminal region has a transcriptional activity (Xia et al., 2010b), and TaNAC8 protein positively regulates wheat defense response to *Pst* (Xia et al., 2010b). During wheat-*Pst* incompatible interaction, *TaNAC8* expression was significantly upregulated at 24 hpi, with no significant increase in expression during compatible interactions. Also, the *TaNAC8* expression was upregulated by methyl jasmonate and ethylene treatments suggesting its involvement in wheat defense response against *Pst* through the ethylene/methyl jasmonate-dependent signal transduction pathway (Xia et al., 2010b). Furthermore, during wheat-*Pst* incompatible interaction, microscopic studies established that the formation of *Pst* haustorial mother cells and haustorium occurred at 18-24 hpi, suggesting that *TaNAC8* is key in defense response for signal transduction (Xia et al., 2010b).

TaNAC21/22 binds ta-miR164 in the nucleus and functions as a transcriptional activator; and silencing this gene reduces wheat stripe rust resistance (Feng et al., 2014). *TaNAC1*, a novel NAC member of the NAC1 subgroup, negatively regulates plant disease resistance and may alter jasmonic acid- and salicylic acid-signaling defense signals in wheat (Wang et al., 2015a). Zhang and colleagues demonstrated that silencing *TaNAC2* increases resistance to various pathogens (Zhang et al., 2018b), and similar results were also reported by Wang and colleagues who found that *TaNAC30* was a negative regulator of wheat resistance to *Pst* isolate CYR31 (Wang et al., 2018). Knockdown of *TaNAC30* gene resulted in improved resistance to *Pst*, but also resulted in a substantial accumulation of H_2_O_2_ (Wang et al., 2018). When the wheat line Thatcher+Lr14b (TcLr14b) was challenged with a *Pt* virulent isolate, *TaNAC35* gene was found to negatively regulate leaf rust resistance (Zhang et al., 2021b). Histological studies showed that silencing *TaNAC35* lowered haustorial mother cell formation and mycelial proliferation, suggesting that this gene is a negative regulator of defense response of wheat line TcLr14b to *Pt* pathotype THTT in a compatible interaction (Zhang et al., 2021b). *TaNAC069* was found to positively regulate wheat resistance to *Pt* infection by activating pathogenesis-related genes and suppressing ROS scavenging-related genes (Zhang et al., 2021c). Another transcription factor, *TaBZR2*, binds to the promoter region of the chitinase gene *TaCht20.2*, resulting in increased chitinase activity, thereby conferring broad spectrum resistance to the stripe rust fungus (Bai et al., 2021). *TuNAC69* significantly contributed to immune response mediated by NLR protein YrU1, and it was anticipated to confer resistance to other pathogens (Xu et al., 2022). Still unanswered questions include how plants transmit immune signals to *TuNAC69* to regulate transcriptional reprogramming in defense responses, and which genes are *TuNAC69*’s direct target which contribute to *YrU1*-mediated resistance and basal immunity (Xu et al., 2022).

### 5.4 Regulatory role of transcription factors in responses to abiotic stresses in wheat

Abiotic stress is one of the most important variables affecting plant growth, development, and production globally. In the recent past years, the use of transgenic approaches led to a significant progress in the identification of key regulators of drought tolerance in wheat. Many transcription factor families have been shown to have a function in plant stress responses. Differential expression of cytochrome P450, glutathione transferase, dehydrins, proteinase inhibitors, heat shock proteins, and regulatory proteins such as transcription factors is a frequent response to abiotic stresses. Several transcription factors, including bHLH, bZIP, ERF, HD-ZIP, NAC, and WRKY, were shown to be differentially expressed in a drought-tolerant wheat genotype compared to a susceptible genotype (Ergen et al., 2009). Transcriptomic and proteomic analyses of a drought-stressed pale green durum wheat mutant revealed expression modulation of many genes encoding photosystem components, antioxidant enzymes, and enzymes involved in carbohydrate metabolism and the tricarboxylic acid cycle, which may be useful in addressing drought resistance in wheat (Peremarti et al., 2014). In wheat, six novel heat-induced *MYB* genes were identified and *TaMYB80* was found to confer heat and drought tolerance in transgenic *Arabidopsis* (Zhao et al., 2017). These findings add to our knowledge of the roles of heat-induced *MYB* genes and serve as the foundation for identifying the best candidates for in-depth functional research of heat-responsive *MYB* genes in wheat. Abiotic and biotic stresses lead to rapid upregulation of ethylene response factors (He et al., 2012), and they have been the focus of many overexpression experiments to determine their use in enhancing drought tolerance. Overexpression of wheat *TaERF1* activated stress-related genes, including pathogenesis-related genes, and enhanced drought, cold, and salt tolerance in transgenic plants (Xu et al., 2007). Overexpression of *TaERF3* in wheat improved drought and salinity tolerance (Rong et al., 2014), possibly due to increased accumulation of proline and chlorophyll content compared to non-transformed lines and activation of several downstream genes by binding to GCC-box *cis*-elements present in target gene promoter regions (Rong et al., 2014). Recently, *AtERF019* was shown to play a role in drought tolerance, with a phenotype of delayed blooming and maturity under drought stress, suggesting that overexpression of its orthologs might be exploited to provide greater drought tolerance in wheat without sacrificing the seed set (Scarpeci et al., 2017).

*TaNAC69* was found to play a role in the response to abiotic stimuli such as cold, drought, and abscisic acid treatments. The expression of three highly homologous *TaNAC69* genes was upregulated by the aforementioned conditions, particularly drought stress (Xue et al., 2006). *TaNAC69* genes were expressed at high levels in the root in unstressed conditions, in addition to being upregulated by drought. This shows that *TaNAC69* genes are involved not only in drought stress, but also in regular cellular functions of roots (Xue et al., 2006). *TaNAC69* overexpression in transgenic wheat increased dehydration tolerance and improved water usage efficiency (Xue et al., 2011). *TaNAC4* expression was enhanced in response to biotic and abiotic challenges such as high salinity, wounding, and low temperature, indicating that *TaNAC4* serves as a transcriptional activator during biotic and abiotic stress responses in wheat (Xia et al., 2010). *TaNAC47* was differentially expressed in various tissues and was induced by stress treatments such as exogenous abscisic acid, polyethylene glycol, cold, and salt. Surprisingly, overexpression of *TaNAC47* was discovered to trigger the expression of downstream genes and affect various physiological indices, potentially allowing transgenic plants to resist adverse environmental conditions. These findings suggest that the wheat *TaNAC47* gene plays a crucial role in response to abscisic acid and abiotic stresses (Zhang et al., 2016a). *TaNAC2* was shown to be implicated in the response to drought, salt, cold, and abscisic acid treatment based on gene expression profiling. *TaNAC2* overexpression in *Arabidopsis* resulted in increased tolerance to salt, drought, and cold conditions, as well as increased expression of abiotic stress-response genes and various physiological markers indices (Mao et al., 2012). The integration of the wheat genome sequence, with transcriptome, proteome, and metabolome profiling of genes associated with various drought-tolerant traits, will help in overcoming the challenges posed by the complexity of the genome and will make it easier to analyze the genetic basis of drought tolerance in wheat. In addition to this, it will aid in the integration of phenotypic, biochemical, and genomics-assisted selection approaches for enhanced breeding of drought-resistant wheat cultivars.

## 6 Non-coding RNAs regulate the resistance against wheat rusts

Non-coding RNAs (ncRNAs) consists of an array of different RNAs. Housekeeping and regulatory ncRNAs are the two main classes of ncRNAs. ncRNAs used to be regarded as trash DNA, but now they are an important part of a variety of regulatory processes (Urquiaga et al., 2021; Waheed et al., 2021). Regulatory ncRNAs can be classified according to their length into small RNAs (siRNAs) and long non-coding (lncRNAs) that only generate small peptides without being translated into proteins (Amor et al., 2009; Urquiaga et al., 2021; Waheed et al., 2021).

### 6.1 sRNAs in wheat defense against rust infection

Immune responses of plants are strongly regulated by an array of immunity-associated regulators like sRNAs and some transcription factors. sRNAs can be classified into three categories based on their biogenesis and structural features and these classes are: short-interfering RNAs (siRNAs), dicer-independent microRNAs (miRNAs) and dicer-independent piwi interacting RNAs (piRNAs) (Chapman and Carrington, 2007; Axtell, 2013; Dubey et al., 2019). sRNAs and miRNAs are similar in size and range between 18–30 nucleotides in length but vary in biogenesis, precursor structures, and mode of action (Waheed et al., 2021). siRNAs are produced from the genome’s hairpin-structured or double-stranded RNA (dsRNA), with the help of RNase II-like endonucleases called dicers, but this does not follow the canonical way towards protein translation (Bartel, 2004; Waters and Storz, 2009). siRNAs are referred to as a subcategory of RNA molecules that play key roles in the diverse strategies that help in imitating, adaptation or suppressing the immune system of the host (González Plaza et al., 2016), alongside other molecules partaking in this multifaceted process. This implies that, the fundamental siRNA pathway components and other various sRNAs function as critical gene expression regulators to fine-tune the immunity of some cereal plants like wheat and rice against pathogen invasion. miRNAs play a crucial role in gene expression regulation *via* chromatin methylation, translational inhibition, or mRNA cleavage (Yu et al., 2017). Various miRNAs associated with different abiotic and biotic stresses were identified and characterized in durum and bread wheat (Alptekin et al., 2017). In wheat, the differential expression of miR159, miR164, miR167, miR171, miR444, miR408, miR1129, and miR1138, a group of miRNAs found in stem rust-infected wheat, appeared to have a regulatory role over *R* genes (Gupta et al., 2012). The role of pathogen-responsive miRNAs in the fine regulation of resistance genes, particularly NBS-LRRs, and PR proteins was demonstrated in some recent studies (Gupta et al., 2012; Kumar et al., 2017). According to the findings, accumulation of miRNAs during the early stages of infection may play a critical role in the host’s hypersensitive response, which decreases as the disease progresses. The differential expression of these miRNAs in the presence and absence of the *R* gene gives a plausible explanation for the distinct pathways mediated by miRNA-controlled *R* genes (Gupta et al., 2012). Twenty-two differentially expressed miRNAs were identified between wheat resistant and susceptible near-isogenic lines inoculated with *Pt.* Upregulation of most miRNAs occurred in susceptible near-isogenic lines compared to resistant near-isogenic lines. This study unraveled the insight into the potential involvement of miRNAs in leaf rust pathogenesis and their wheat target genes (Kumar et al., 2014b). *NB-LRR* defense genes were reported to target some five miRNA families (Zhang et al., 2019). Multiple types of *NB-LRR* genes are regulated by these miRNAs, the majority of which have coiled-coiled domains, and cause secondary siRNAs formation in the target site in a phased pattern. This shows that non-conserved miRNAs, which control disease resistance genes in gymnosperms and angiosperms, display fast flexibility in sequence variants, gene copy number, functions, and expression level (Zhang et al., 2019). Trans-acting-small interfering RNAs (Ta-siRNAs) were also found in wheat (Dutta et al., 2017). Comparative expression analysis of TAS, ta-siRNAs, and their target genes showed a differential and reciprocal relationship as well as discrete patterns between resistant and susceptible near-isogenic lines. The expression profiles of the target genes of the identified ta-siRNAs advocate more towards ETS favoring pathogenesis (Dutta et al., 2017). However, the mechanisms by which ta-siRNAs influences pathogenesis remains a mystery.

### 6.2 lncRNAs in wheat defense against rust infection

Plants use ETI-driven programmed cell death as an efficient defense response since obligate biotrophic fungi require living plant cells for sustenance (Mendgen and Hahn, 2002). These defense responses need rigorous gene expression regulation in the background, which allows the accomplishment of significant transcriptional reprogramming in the infected plant (Bhatia et al., 2021). lncRNAs involvement in the regulation of plant defense mechanisms against obligatory biotrophs has only recently been explored in depth, with a few of studies having been carried out so far (Zhang et al., 2020a). Even so, the majority of the studies in plants focus on hemi-biotrophic fungal pathogen-responsive lncRNAs. Following these studies, Zhang and colleagues carried out a genome-wide analysis of long intergenic ncRNAs (lincRNAs) in a wheat line with great resistance to *Pst*, and 52 lincRNAs were shown to be highly expressed in response to stripe rust infection (Zhang et al., 2016b). As part of the research to better understand their regulatory activities, miRNA target sites were anticipated, with 5 lincRNAs being identified as probable targets and endogenous target mimics (eTM) of miRNAs. lincRNAs can function as eTMs, allowing miRNAs to rescue their intended targets of miRNAs (mRNAs). This showed that lincRNAs can play a more complex function in the regulation of miRNAs, rather than simply serving as their precursor molecules. It was concluded that, the interactions between lincRNAs, miRNAs, and their corresponding mRNAs may regulate plant responses to *Pst* (Zhang et al., 2016b). However, when compared to functional gene expression, the identification of lincRNAs in wheat is still in its early stages. Future studies should focus on the mechanisms exploited by lincRNAs in response to different biotrophic pathogens as well as other biotic and abiotic factors.

## 7 Conclusion and future perspectives

Without a doubt, rust diseases continue to threaten present and future maximization of wheat yields. Plants have evolved a complex network of biochemical pathways, some of which respond to fungal infection and colonization. Recent advances in wheat genome, pan-genome sequencing, mutant genomics, gene capture, and high throughput genomics technologies such as genome editing and gene cloning are enhancing more insights into the interactions between wheat and rust pathogens. A wide range of resistance gene classes, including receptor kinases, are anticipated to be targeted in the future by mutational genomics tools such as MutRenSeq. The identification of novel *R* genes is increasing our understanding of plant innate immunity beyond classic ETI, even though experimental confirmation of some postulated pathways is still pending. The recent breakthroughs in the discovery of resistosome formation by NLRs in *Arabidopsis* made significant contributions to our knowledge of NLR function at molecular level. However, neither the *Lr, Yr*, nor *Sr* genes have been subjected to comparative research involving the formation of resistosomes, as have the other genes. Consequently, this is a crucial issue for further investigation in the future. Rust pathologists and the wheat breeding community’s focus has shifted towards the identification and use of non-race specific adult plant resistance genes for durable resistance because of the emergence of new rust fungi races and the quick inefficacy of race-specific genes. Combining race-specific resistance genes with non-race specific genes is the most promising deployment strategy for minimizing pathogen virulence evolution and ensuring resistance persistence. Such resistance gene pyramids might be created using conventional breeding methods such as marker-assisted selection based on cloned gene sequences or by deploying resistance gene cassettes that incorporate many cloned genes into a single locus. Translational research on gene stacks may give also an answer for biotrophs, and specific breeding strategies based on resistance gene toolkits may enhance long-term disease resistance strategies. A number of proof-of-concept experiments have shown that gene stacking and genome editing may be utilized to develop broad-spectrum disease resistance. Incorporating a five-gene cassette *Sr22–Sr35–Sr45–Sr50–Sr55* into wheat to achieve broad spectrum resistance against stem rust exemplifies the potential of enhancing durable resistance (Luo et al., 2021). Understanding the potential for additive interactions between *R* genes is critical for determining the most successful combinations to pursue, while discovering rust *Avr* genes is also vital for monitoring pathogen development and prioritizing *R* genes for deployment. In-depth research has been conducted on transcriptional regulatory variables associated with disease resistance, as well as the regulatory mechanism of sRNAs in rust resistance. The development of wheat cultivars with enhanced characterization, including pathogen stress requires basic knowledge about physiological, gene regulatory, and biochemical networks. The elucidation of the transcriptional reprogramming and multifaceted mechanisms involved in defense responses can be enhanced by integrating experimental and bioinformatic approaches. Deciphering the roles of different transcription factors in defense responses necessitates functional analysis and molecular characterization based experimental approaches. This will ultimately help in genetic engineering and resistance breeding against rust pathogens. Future breeding projects should also aim to identify exploited and unexploited *R* genes for each disease and the combination of these genes for the generation of numerous pathogens-resistant varieties by CRISPR-Cas9 or classical breeding technology.

Although great gains have been achieved in the research of interaction between pathogen and host, however, we have failed to manage rusts because of ignoring that outcomes of disease resistance are highly dependent on agronomic and environmental factors either reported earlier or need further research. Considering the significant contribution of different abiotic and biotic factors in the development of plant disease epidemics, modeling tools need to be strengthened for precise and timely prediction of possible changes occurring in the agro-climate scenarios as influenced under changing climate and its effect on different host-pathosystems and impact assessment. Exhaustive coordination is required among the researchers from different disciplines like plant pathologists, agronomists, climatologists, epidemiologists, computer scientists, and agro-meteorologists, to further streamline the future work related to the effect of climate change on fluctuating severity, prevalence, and distribution of wheat diseases and shift in the pathogen population. Future climate change research should primarily focus on minimizing the harmful effects of both biotic and abiotic stresses on plant growth and health, and generating inclusive and pertinent prediction model (s) to predict the effect of changing climate on wheat health and productivity in the future. Future resistance breeding strategies will need to be modified to account for the long-term shifts in disease incidence, placing a premium on stability and longevity of disease resistance in the face of heat and water stress. This review serves as a reference point for molecular plant pathologists to better understand the complexities of these diseases and to approach them in a more holistic way.

## Author contributions

Conceptualization, JM and WY; literature search, JM and WY; writing—original draft preparation, JM; writing—review and editing, JM, WY, NZ, LZ, WL, and JC; supervision, WY; funding acquisition, WY. All authors contributed to the article and approved the submitted version.

## Funding

This work was funded by the Natural Science Foundation of China (No. 301871915, 32172367), Natural Science Foundation of Hebei Province (C2020204071), Modern Agricultural Industry System of Wheat Industry in Hebei Province (No. HBCT2018010204).

## Conflict of interest

The authors declare that the research was conducted in the absence of any commercial or financial relationships that could be construed as a potential conflict of interest.

## Publisher’s note

All claims expressed in this article are solely those of the authors and do not necessarily represent those of their affiliated organizations, or those of the publisher, the editors and the reviewers. Any product that may be evaluated in this article, or claim that may be made by its manufacturer, is not guaranteed or endorsed by the publisher.
